# Propagation of PrP^Sc^ in mice reveals impact of aggregate composition on prion disease pathogenesis

**DOI:** 10.1038/s42003-023-05541-3

**Published:** 2023-11-14

**Authors:** Sheng Chun Chang, Samia Hannaoui, Maria Immaculata Arifin, Yuan-Hung Huang, Xinli Tang, Holger Wille, Sabine Gilch

**Affiliations:** 1 Department of Comparative Biology and Experimental Medicine, Faculty of Veterinary Medicine, University of Calgary, Calgary, AB Canada; 2 Hotchkiss Brain Institute, Cumming School of Medicine, University of Calgary, Calgary, AB Canada; 3 Department of Biochemistry, Center for Prions and Protein Folding Diseases, University of Alberta, Edmonton, AB Canada; 4 Neuroscience and Mental Health Institute, University of Alberta, Edmonton, AB Canada

**Keywords:** Neurodegeneration, Pathogens, Biochemistry

## Abstract

Infectious prions consist of PrP^Sc^, a misfolded, aggregation-prone isoform of the host’s prion protein. PrP^Sc^ assemblies encode distinct biochemical and biological properties. They harbor a specific profile of PrP^Sc^ species, from small oligomers to fibrils in different ratios, where the highest infectivity aligns with oligomeric particles. To investigate the impact of PrP^Sc^ aggregate complexity on prion propagation, biochemical properties, and disease pathogenesis, we fractionated elk prions by sedimentation velocity centrifugation, followed by sub-passages of individual fractions in cervidized mice. Upon first passage, different fractions generated PrP^Sc^ with distinct biochemical, biophysical, and neuropathological profiles. Notably, low or high molecular weight PrP^Sc^ aggregates caused different clinical signs of hyperexcitability or lethargy, respectively, which were retained over passage, whereas other properties converged. Our findings suggest that PrP^Sc^ quaternary structure determines an initial selection of a specific replication environment, resulting in transmissible features that are independent of PrP^Sc^ biochemical and biophysical properties.

## Abstract (web summary)

Prion aggregate complexity affects prion biochemical properties, neuropathology, and clinical signs upon infection of mice. Aggregate size-specific clinical signs are retained upon passage despite convergence of prion properties.

## Introduction

Prion diseases are invariably fatal and infectious neurodegenerative diseases affecting humans and various animals^1^. The causative agents of these diseases are prions, proteinaceous infectious particles that replicate without using nucleic acids. Prions arise by the misfolding of the host-encoded membrane glycoprotein PrP^C^ into the pathogenic isoform PrP^Sc^ ^2^. In contrast to PrP^C^, which is mainly alpha-helical, PrP^Sc^ contains a beta sheet-rich structure with a core resistant to proteases and is prone to aggregation^3^. In prion propagation, PrP^Sc^ acts as a template, or seed, likely in conjunction with host co-factors^4^, to promote the structural conversion of PrP^C^ into newly generated PrP^Sc^. This self-replicating process leads to the deposition of pathogenic PrP^Sc^ assemblies in the brain and some extraneural tissues^2^. The converted subunits become part of a growing chain that may take on various aggregation states ranging from few molecules to oligomers to amyloid fibrils with various infectious efficiencies^5–9^. Different instances of prion disease in the same host may be characterized by different clinical and pathological features with PrP^Sc^ harboring varying biochemical properties, which are stable upon passage. These observations provided meaning to the concept of prion strains, in which the existence of different PrP^Sc^ conformers gives rise to different phenotypes of prion diseases^10–16^.

Similar to biochemical properties of PrP^Sc^, such as PrP^Sc^ electrophoretic mobility, conformational stability and/or protease resistance, and glycoform ratios^10–14^, the PrP^Sc^ aggregate composition, i.e., the relative distribution across different quaternary structures (multimeric PrP^Sc^ assemblies with specific tertiary structures), from oligomers to fibrils, is considered strain specific^17,18^. A pioneering study by Silveira et al.^19^ used asymmetrical flow field-flow fractionation to separate purified and proteinase K-treated 263K prions into PrP^Sc^ particles according to size. They were able to link prion particle size, i.e., quaternary structure variations, to infectivity, showing that the most infectious prion particles adopt a non-fibrillar, oligomeric organization that correspond to the size of 14-28 PrP molecules^19^. The study also revealed, with respect to infectivity and converting activity that large fibrils were less active, and oligomers equal or smaller than 5 PrP-mers were lacking any activity, at least, in the context of 263K prion strain. Other studies used sedimentation velocity ultracentrifugation to separate PrP^Sc^ particles of various rodent-adapted prion strains, notably with detergent-solubilized brain homogenates without PK digestion^17,18,20,21^. The size distribution of PrP^Sc^ particles was correlated to infectivity in transgenic mouse models^17,18^. In these studies, the properties of the prion particles with highest infectivity were greatly varying in size, and were determined to be strain-specific, but in general adopt an oligomeric or small oligomeric profile^17,18^. More recently, the same group has investigated additional implications of prion quaternary structures. The replication kinetics relevant to generating different PrP^Sc^ aggregates appear to happen within two distinct phases in the early stage of replication, and this event is independent of the prion strain, where smaller aggregates are generated during the first process of formation in the early stages of disease, and they are transformed into larger aggregates in the second process^22^. Furthermore, the molecular aspects of the species barriers regarding PrP conversion have been unveiled in another study^23^. Interestingly, fractionating prion inoculum has been shown to highly increase the species barrier, while unfractionated prions, where all different aggregates are present, seem to be crucial in crossing the species barrier and thus, inducing disease^23^. In another recent study, distinct PrP^Sc^ size distribution profiles and seeding activities were correlated with the disease phenotypes of hamster prion strains inducing disease after long or short incubation periods, and oligomeric PrP^Sc^ was neither observed as the predominant conformer in the fast strains, nor the size with the greatest seeding activity^24^.

Chronic wasting disease (CWD), a prion disease that affects free-ranging and farmed cervids, is considered the most contagious form of prion disease. The rapid spread of CWD in North America and Northern Europe^25–27^ is in part due to the significant quantity of infectivity in peripheral tissues and excreta that are released into the environment and contribute to lateral transmission of disease^28–43^. Even more concerning, recent experimental studies indicate that the transmission barrier of CWD to humans is not absolute^44–47^. To date, a number of CWD strains have been identified^48–55^, however, studies have remained relatively rare with regards to understanding the actual number of CWD strains circulating naturally in the field and their ability to evolve within and adapt to other species.

The structural diversity of prion assemblies and their correlation to pathogenicity are fundamental in understanding the principles of prion replication and spreading processes. However, there is a lack of information to date for non-rodent-adapted prions, and knowledge about PrP^Sc^ particle size as a contributor to disease phenotype and pathology is limited.

**Fig. 1:**
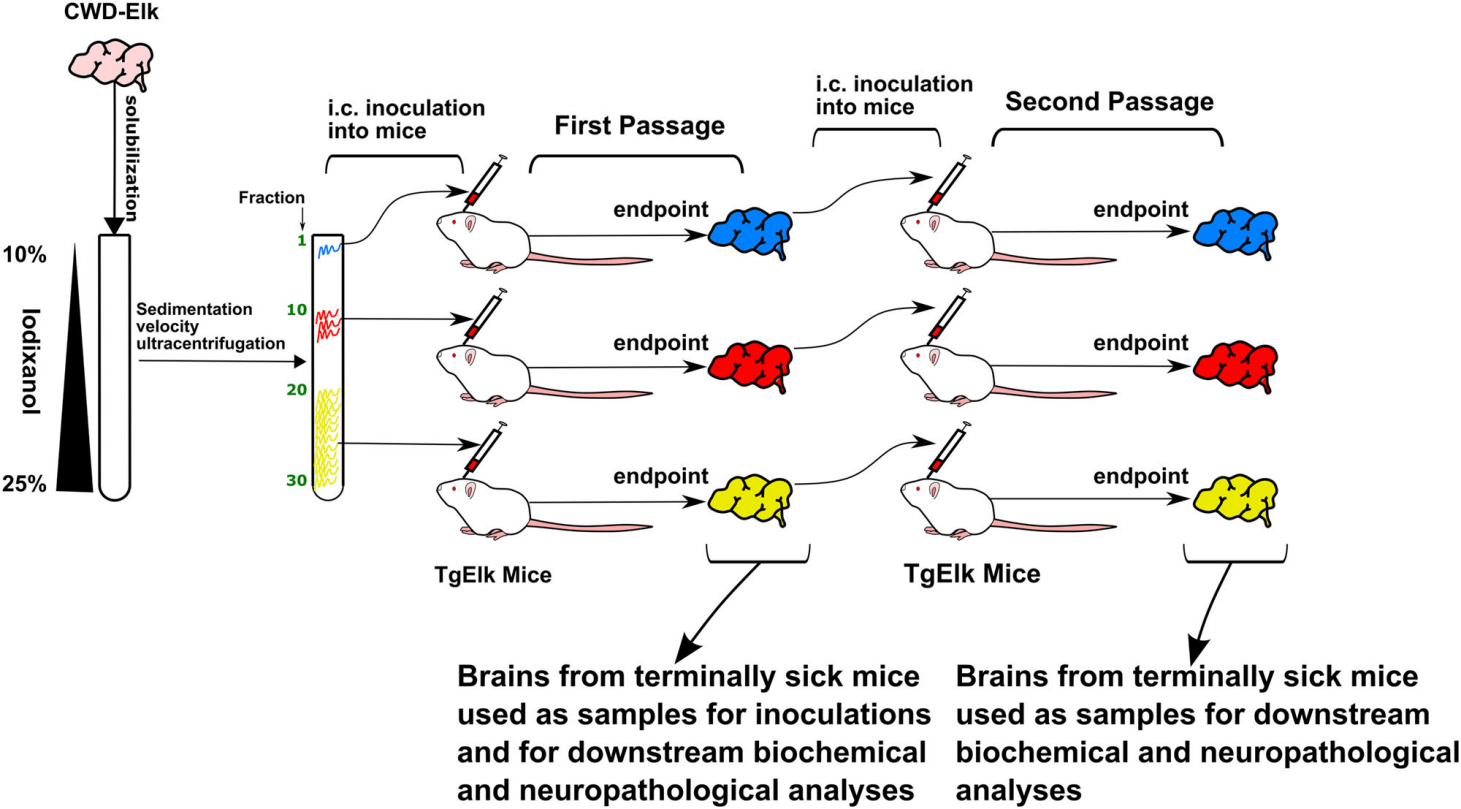
Schematic outline of the study. Homogenized and solubilized CWD-Elk prion brain samples were subjected to sedimentation velocity ultracentrifugation that results in the smaller aggregates to be found near the top, medium-sized aggregates to be found in the middle, and large aggregates to be found near the bottom of the gradient. Even fractions, were intracerebrally inoculated into transgenic mice expressing elk PrP^C^ and their brains were harvested at endpoint. These samples, denoted first passage, were used for downstream experiments, namely PK digestion, sedimentation analysis, ELISA, histopathology, and immunohistochemistry (Figs. 3–6). The samples were also further inoculated into transgenic mice, and these animals’ brains were harvested at endpoint. These subsequent samples, denoted second passage, were used for downstream experiments (Figs. 4, 6–9).

We used sedimentation velocity centrifugation to fractionate and separate different assemblies of PrP^Sc^ according to their quaternary structure. We used elk CWD prions originated from an experimental pathogenesis study, where elk were orally challenged with brain homogenate from a CWD-positive farmed elk. Prions from the experimental inoculation were characterized as CWD2 strain^48,56^. We inoculated transgenic mice overexpressing elk PrP^C^ (tgElk^57^ Fig. 1) with these fractions. We found that infectivity of CWD PrP^Sc^ aggregates neither correlates with the amount of PK-resistant PrP^Sc^ detectable in the individual fractions nor that of previously studied rodent-adapted scrapie strains^18^. The size of PrP^Sc^ aggregates determined survival times and clinical signs in tgElk mice as well as PrP^Sc^ levels generated upon first passage and their biochemical properties, aggregate profiles, and distribution within the brain. Upon passage, different clinical signs were retained even though biochemical, biophysical, and neuropathological profiles as well as survival times, converged. Our data suggest that PrP^Sc^ aggregate size might be a determining component to achieving the most efficient propagation of disease-inducing PrP^Sc^ species in the brain. PrP^Sc^ quaternary structures appear to be directly linked to the different clinical signs observed in tgElk mice, a feature that is retained upon passaging.

## Results

### PrP^Sc^ aggregate size affects infectivity and clinical signs in vivo

**Fig. 2:**
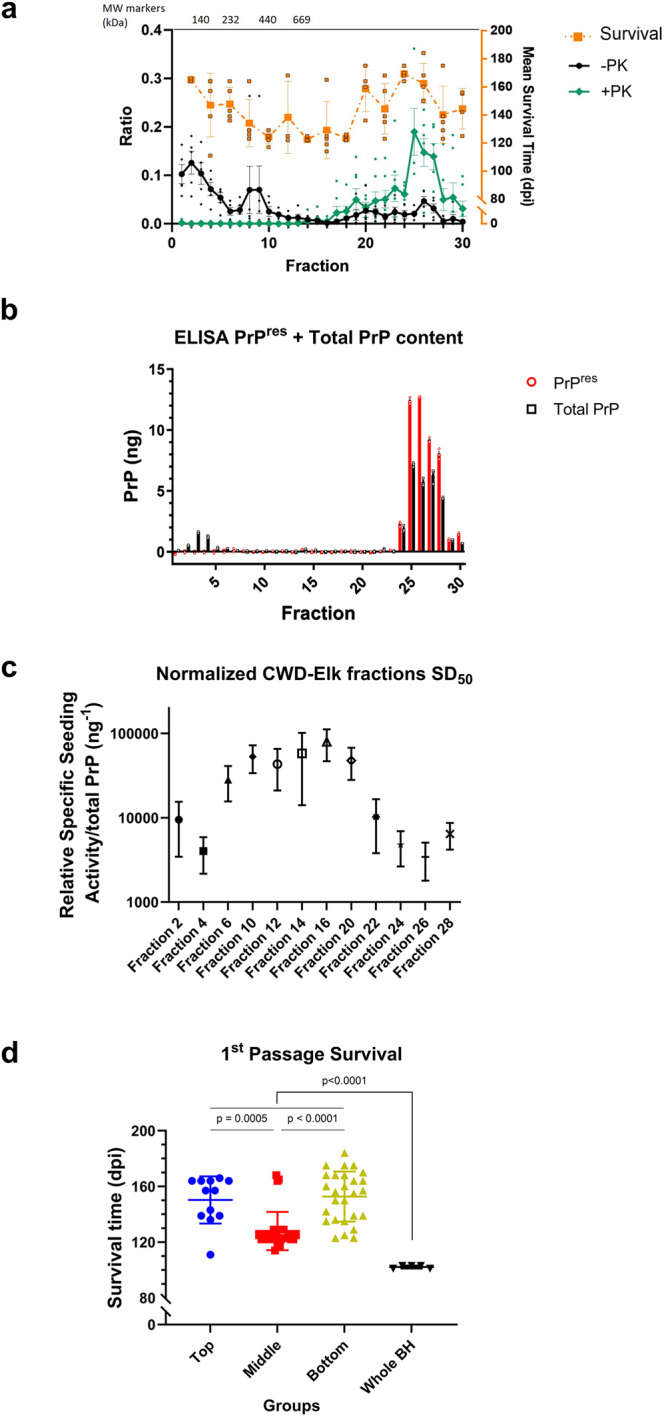
Sedimentation velocity gradient and bioassay in tgElk mice. CWD-Elk prions were solubilized and fractionated through sedimentation velocity ultracentrifugation, and alternating fractions (even-numbered) were inoculated into tgElk mice. **a** Fractions collected were quantified by Western blot for total PrP (-PK; black line) or PrP^res^ (+PK; green line) (mean ± SEM; -PK n = 5, +PK n = 7 independent experiments), and for survival times of inoculated tgElk (orange line) (mean ± SD; n = 2 for fraction 2, n = 3–5 for other fractions). Western blot signals in each fraction from each replicate are calculated as a ratio of the sum of all signals from the respective replicate. **b** ELISA quantification of total PrP (black) or PrP^res^ content (red) (mean ± SEM; n = 3 replicates). **c** SD_50_/ng PrP contained in each fraction (mean ± standard error, n = 8–12 replicates across 3 independent experiments). **d** Survival times of the tgElk mice inoculated with fractions from the top (blue), middle (red), or bottom (green) of the gradient, or unfractionated CWD-Elk brain homogenate. Each point corresponds to the survival of an individual animal. Mean ± SD; top group n = 12, middle group n = 28, bottom group n = 27, whole BH group n = 5 animals.

In order to characterize the PrP^Sc^ aggregate profile and infectivity of prions derived from the natural host species, we subjected CWD-infected elk brain homogenates characterized as CWD2^48^ to sedimentation velocity centrifugation (Fig. 1). Thirty fractions were collected, with fraction 1 at the top and fraction 30 at the bottom (Fig. 1). To determine the PrP content within each fraction, we quantified the levels of total PrP (-PK) and PK-resistant PrP^Sc^ (+PK; PrP^res^) by Western blot (Supplementary Fig. 1; Fig. 2a, black and green lines respectively) and ELISA (Fig. 2b). We found that fractions 1-9 contained the highest levels of total PrP that was PK-sensitive. Fractions 10-17 had the lowest total PrP and PrP^res^ content at almost undetectable levels, while fractions 18–30 of the gradient contained exclusively PrP^res^, with peak signals in fractions 25–27. To further characterize the properties of PrP^Sc^ multimers of different fractions, we used real-time quaking-induced conversion (RT-QuIC) assay to determine the 50% seeding dose, SD_50_/ng of total PrP (quantified by ELISA) for individual fractions. We found that PrP content of different groups of fractions did not correlate with seeding activity. SD_50_/ng of PrP were highest in fractions 6–20 and reduced in fractions of lower (fractions 2 and 4) or higher (fractions 22–28) molecular weight (Fig. 2c). Next, we inoculated even-numbered fractions into transgenic mice overexpressing elk PrP^C^ (tgElk)^57^. Interestingly, we observed that the tgElk mice presented with different clinical signs, depending on the fraction used for inoculation. Animals inoculated with the top fractions (2-6) generally exhibited signs of hyperexcitability, while animals inoculated with bottom fractions (20–30) generally exhibited signs of lethargy. Mice inoculated with the middle fractions (8–18) were neither hyperexcitable nor lethargic, and they did not exhibit clinical signs that were specific to these fractions.

Similar to previous results from our group^58^, tgElk mice inoculated with unfractionated CWD-Elk brain homogenate had an average survival of 102 days post-inoculation (dpi; Fig. 2d). Amongst mice inoculated with different fractions, we observed the shortest survival of 128 ± 14 dpi among animals inoculated with fractions 8–18 (Fig. 2d). TgElk mice inoculated with fractions 2-6 (top of the gradient), containing high amounts of total PrP, or fractions 20–30 (bottom of the gradient) composed mainly of PrP^res^, had longer average survival times of 150 ± 17 and 153 ± 18 dpi, respectively (Fig. 2d).

To analyze whether the levels of prion seeding activity/infectivity were saturated in the fractions, we performed a serial protein misfolding amplification assay (sPMCA), which recapitulates prion propagation in a specific and sensitive manner and generates infectious prions^59^. We tested the propagation of selected fractions representing low (fractions 4 and 6), medium (fractions 14 and 18), and high (fractions 26 and 28) molecular weight PrP^Sc^. Seed dilutions between 10^−2^ and 10^−4^ were amplified over three rounds of PMCA (Supplementary Fig. 2). In all fractions, PrP^res^ generated in round 1 was absent or weak (e.g., fraction 14; Supplementary Fig. 2a). In round 3, PrP^res^ was generated in all fractions and dilutions, with the weakest amplification observed in reactions seeded with fractions 4 and 6 (Supplementary Fig. 2). Most importantly, these results indicate that the overall level of prion seeding activity/infectivity in any of the representative fractions, used for inoculations, was below saturation.

In summary, these results demonstrate that the SD_50_/ng of PrP in RT-QuIC correlates with levels of infectivity, which were both highest in the middle fractions (8–19); however, there was no direct correlation with the total amount of PrP per fraction, as the middle fractions contained lower amounts of total PrP than the top fractions and lower amounts of PrP^res^ than the bottom fractions.

### Prion protease resistance, quantity, and sedimentation properties depend on PrP^Sc^ aggregation state of inoculum

**Fig. 3:**
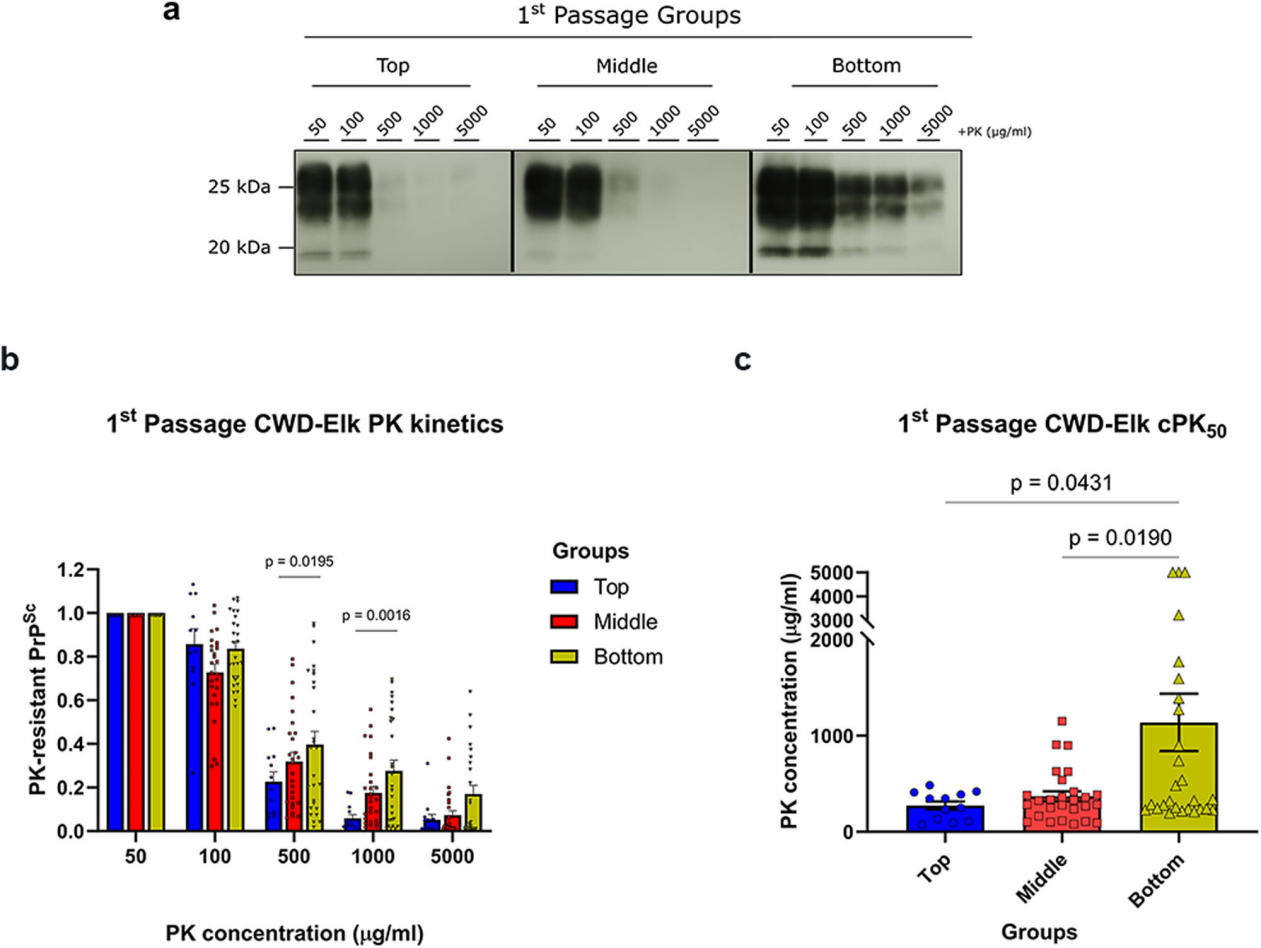
Biochemical analyses of tgElk brain homogenates inoculated with CWD-Elk fractions (first passage). **a** Representative blots of PrP^Sc^ digested with various concentrations of PK from the top, middle, and bottom groups from the first passage. **b** Densitometric analysis of the PrP^res^ signals. Signals were quantified as a ratio of the baseline signal at 50 μg/ml. Statistical analysis was performed with two-way ANOVA followed by Tukey’s post-hoc multiple comparison test. **c** cPK_50_ analysis of the PrP^res^ signals of the three groups. The PK concentration required to degrade 50% of the signal was obtained from the baseline signal of 50 μg/ml digestion. Mean ± SEM; top group n = 12, middle and bottom groups n = 27 replicates with at least three biologically independent samples.

Given the profound differences in survival times depending on fractions used for inoculation, and clinical signs elicited by smaller versus larger CWD prion particles, we asked whether these are accompanied by biochemical and/or biophysical differences of PrP^Sc^ from inoculated tgElk. We first determined the resistance to proteolytic degradation of the PrP^Sc^ from the brains of animals inoculated with the top, middle, and bottom fractions—hereafter referred to as, top, middle, and bottom groups. We found that PrP^Sc^ in the brains of mice from the bottom group was more resistant than that from the top group when digested with 500 μg/ml of PK or higher concentrations (Fig. 3a, b). When we determined the PK concentration needed to degrade 50% of the PrP^res^ signal (cPK_50_) obtained from the baseline PK concentration of 50 μg/ml, we found that the average cPK_50_ of the bottom group was significantly higher at 1139 ± 298 μg/ml than that of the top and middle groups (274 ± 42 and 371 ± 52 μg/ml, respectively; Fig. 3c). This highlights that PrP^Sc^ of different aggregation states, when propagated in vivo, induces PrP^Sc^ with different PK resistance resembling that of the fractions used for inoculation.

**Fig. 4:**
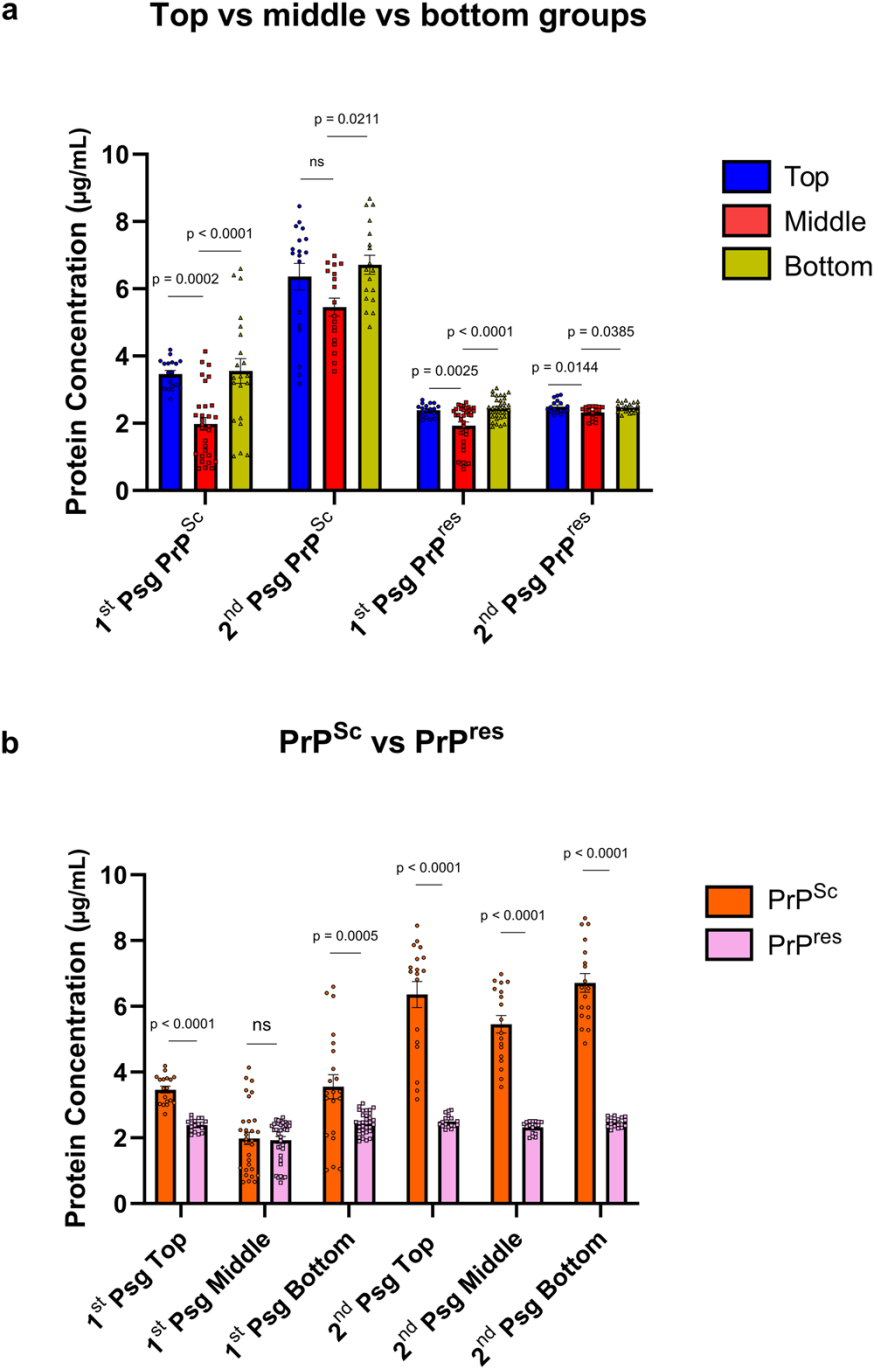
PrP^Sc^ and PrP^res^ levels in the 1st and 2nd passage brain samples quantified using ELISA. **a** PrP concentration compared between top (blue), middle (red), and bottom (green) groups in first and second passages. Statistical analysis was performed using one-way ANOVA followed by *post-hoc* analysis with Tukey’s multiple comparison test. **b** Comparison of levels of PrP^Sc^ (orange) with respect to PrP^res^ (pink) between top, middle, and bottom groups upon first and second passages. Statistical analysis was performed with unpaired Student’s *t*-test. Mean ± SEM; 2nd passage PrP^res^ top and middle groups n = 17; 1st passage PrP^Sc^ top group, all 2nd passage PrP^Sc^ groups, 1st passage PrP^res^ top group, and 2nd passage PrP^res^ bottom group n = 18; 1st passage PrP^Sc^ bottom group n = 21; 1st passage PrP^Sc^ middle group n = 30; 1st passage PrP^res^ bottom group n = 33; 1st passage PrP^res^ middle group n = 36 replicates; all experiments involve at least three biologically independent samples.

Having found that different PrP^Sc^ aggregates can determine the PK resistance of the newly propagated PrP^Sc^ in vivo, we asked whether the aggregation state of the prion inocula may affect the absolute concentration of propagated PrP^Sc^ in the brain, which might be a determining factor in disease pathogenesis. We conducted ELISA, with solubilization and centrifugation steps allowing the elimination of PrP^C^, with or without PK digestion to determine the quantity of PrP^res^ and total PrP^Sc^, respectively, in brain homogenates of mice from all three groups. We found that PrP^Sc^ and PrP^res^ content in the middle group, respectively at 1.98 ± 0.19 and 1.93 ± 0.11 μg/ml, were significantly lower than that of the top and bottom groups (Fig. 4a). Also, the concentration of PrP^Sc^ was significantly higher than that of PrP^res^ in the top and bottom groups but not in the middle group (Fig. 4b).

**Fig. 5:**
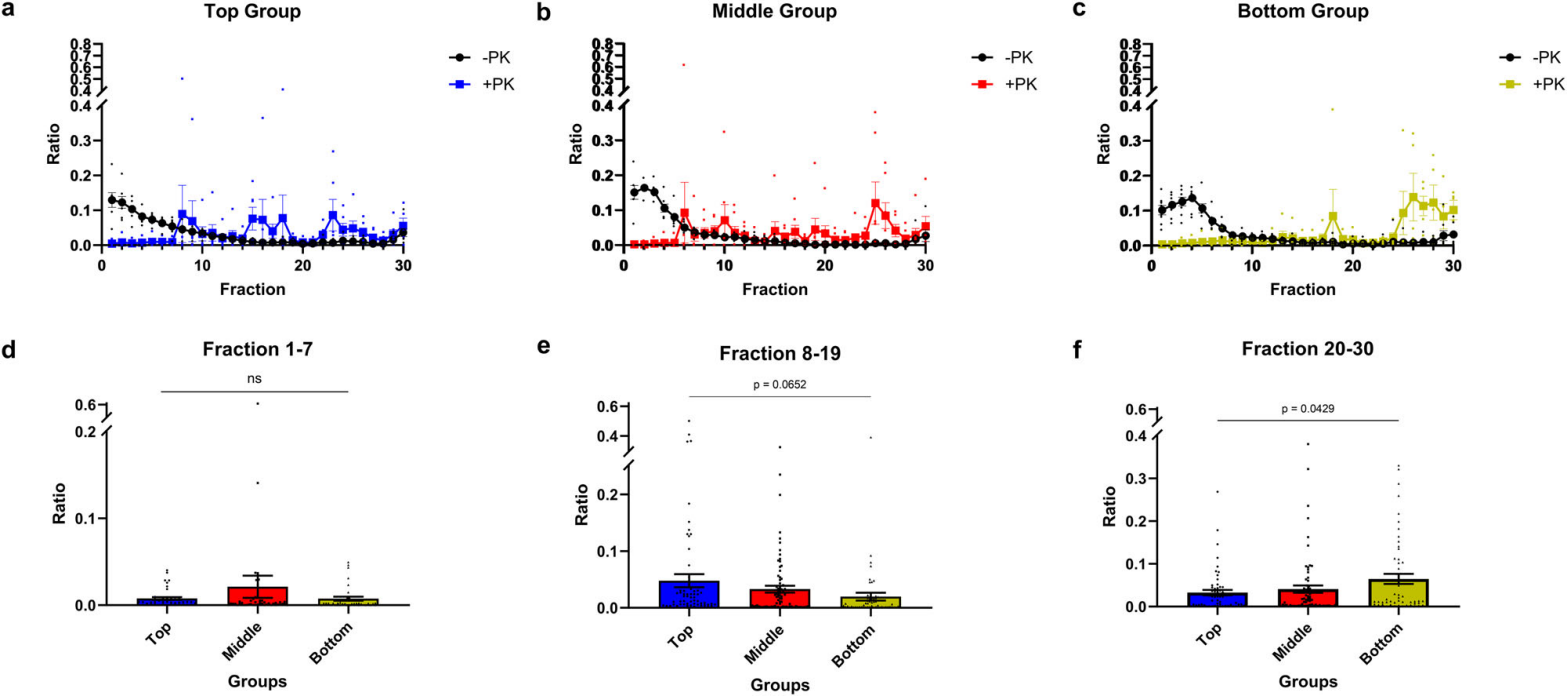
Sedimentation profiles of top, middle, and bottom groups in first passage. Brain homogenates from top (**a**), middle (**b**) and bottom (**c**) groups were solubilized and fractionated by SV. Fractions collected from the gradients were quantified for PrP content (-PK; black line; +PK; blue, red and green, respectively for top, middle and bottom groups). The +PK signals at the top (**d**), middle (**e**), and bottom (**f**) of the gradient denoted respectively as fractions 1–7, 8–19, and 20–30 were depicted as the average signal per fraction in each group to indicate the trend of greater amounts of +PK signals in the top fraction compared to the bottom fractions. Mean ± SEM; n = 5–8 independent experiments with at least three biologically independent samples.

We next sought to establish the physical properties of PrP^Sc^ in the brains of mice inoculated with top, middle, and bottom fractions by characterizing the sedimentation velocity profiles. Brain homogenates from tgElk mice inoculated with fractions of CWD-Elk prions were subjected to sedimentation velocity gradient centrifugation, and PrP signals in the fractions, with and without PK digestion, were quantified using Western blots (Fig. 5a–c). The sedimentation profiles of PrP^res^ within the gradient differ among the three groups. Overall, in brains of mice inoculated with top or middle fractions, PrP^res^ signals were distributed throughout the gradient (Fig. 5a, b). Upon inoculation of bottom fractions, a clear PrP^res^ peak can be observed in fractions 25–30 (Fig. 5c). To quantitatively compare these differences, we determined the average ratios of PrP^res^ signals in top, middle and bottom fractions of the gradients between brain homogenates of inoculated mice (Fig. 5d–f). We observed that there was significantly more PrP^res^ at the bottom of the gradient (i.e., fractions 20–30) in brains of mice inoculated with bottom fractions compared to those inoculated with top fractions. There is also the trend of greater amounts of PrP^res^ in fractions 8–19 in the top fractions inoculated group compared to the bottom fractions inoculated group. This shows that the type of PrP^Sc^ aggregates used for inoculation determines the biochemical and biophysical properties of newly generated PrP^Sc^.

### TgElk inoculated with different PrP^Sc^ fractions have different PrP^Sc^ deposition profiles

**Fig. 6:**
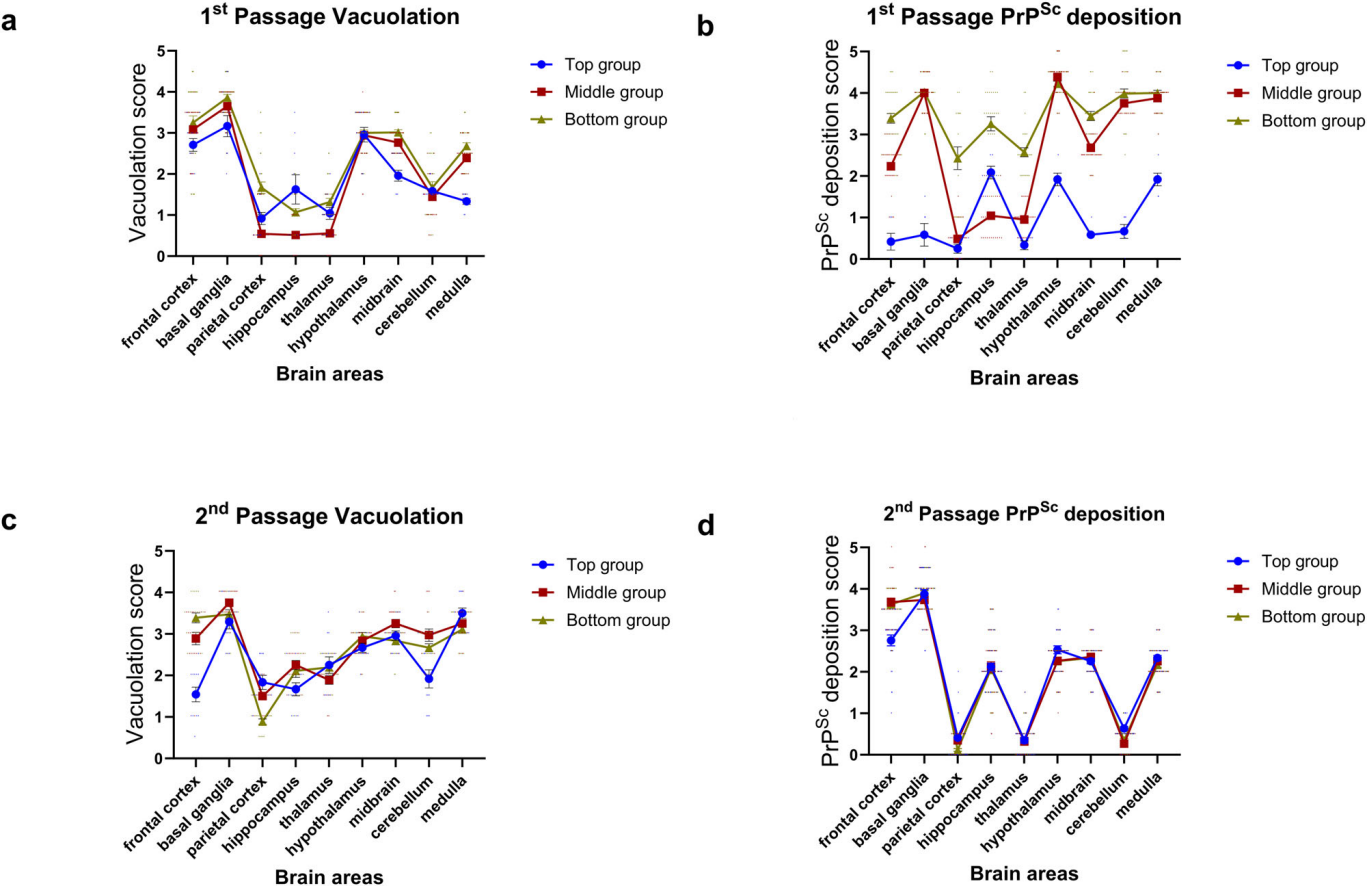
Brain vacuolation and PrP^Sc^ deposition scorings in tgElk mice inoculated with CWD-Elk fractions. Brain vacuolation was semi-quantified (scored) to compare first passage (**a**) to second passage (**c**). The y-axis represents vacuolation scores in a range of 0 (none) to 5 (severe). The x-axis represents the nine brain areas scored. Abnormal PrP deposition was scored to compare first passage (**b**) to second passage (**d**). The y-axis represents scoring of abnormal PrP-deposits on a range of 0 (none) to 5 (severe). The x-axis represents the nine brain areas scored. Mean ± SEM; 1st passage vacuolation top n = 12, middle n = 36, bottom n = 36; 1st passage PrP^Sc^ deposition top n = 6, middle n = 36, bottom n = 36; 2nd passage vacuolation top n = 12, middle = 18, bottom = 18; 2nd passage PrP^Sc^ deposition top n = 33, middle = 39, bottom = 39 individual scores. Scores were derived from a minimum of three biologically independent samples.

To elucidate a reason for the observed differences in clinical signs, we conducted histopathological and immunohistochemical analyses of brain samples of animals inoculated with different fractions. We performed hematoxylin and eosin (H&E) staining on brain slices to determine the degree and distribution of spongiform lesions in brain tissues of animals in different groups (Fig. 6a). There were no significant variations in the vacuolation profiles between the different groups. We then determined the intensity and distribution of PrP^Sc^ deposits that differed for the groups (Fig. 6b). Elevated levels of PrP^Sc^ deposits were generally observed in the brains of mice from the bottom group across all the analyzed brain areas. Low levels of PrP^Sc^ were observed in the top group across all of the analyzed brain areas except for the moderate levels observed in the hippocampus, hypothalamus, and medulla. The middle group had low levels of PrP^Sc^ deposits in the parietal cortex, hippocampus, and thalamus, and elevated deposit levels elsewhere in the analyzed brain areas. In summary, we demonstrate that the type of PrP^Sc^ aggregate used for inoculation can determine the distribution of PrP^Sc^ in the brain.

### Differences in clinical signs are retained upon second passage in tgElk mice

We have shown that PrP^Sc^ aggregation state is a key determinant in the resulting biological, biochemical, and physical properties when propagated in an in vivo CWD model. Since these differential properties are reminiscent of those observed in different prion strains, we tested whether the observed differences would be sustained upon passaging.

**Fig. 7:**
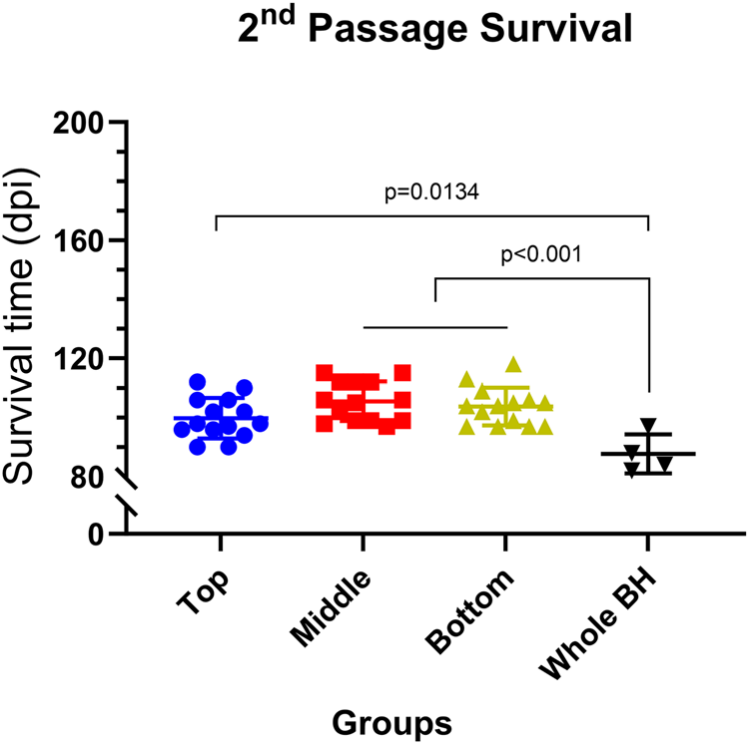
Survival times of tgElk mice upon second passage. TgElk mice were inoculated with the first passage prion material from the top (blue), middle (red), and bottom (green) groups as well as from control unfractionated (whole BH) group. Each point corresponds to the survival of an individual animal. Mean ± SEM; top, middle, and bottom groups n = 14, whole BH group n = 4 animals.

Brain homogenates from mice inoculated with top, middle, and bottom fractions (first passage), as well as from the unfractionated group, were used to inoculate tgElk mice for the second passage. These animals had reduced survival compared to the first passage, at 100 ± 7, 106 ± 7, and 104 ± 6 days, respectively for top, middle, and bottom groups (Fig. 7). With around 50 days, survival time reductions were most pronounced for the top and bottom groups, compared to a reduction of about 20 days in the middle group (Supplementary Fig. 3a). However, the difference in survival times among the three fractionated groups was no longer significant. The survival times in the unfractionated group were significantly less compared to the other groups (Fig. 7). Intriguingly, the clinical signs of hyperexcitability associated with initial inoculation of PrP^Sc^ aggregates from the top of the gradient and lethargy in mice inoculated with aggregates from fractions 20–30 were retained upon second passage.

**Fig. 8:**
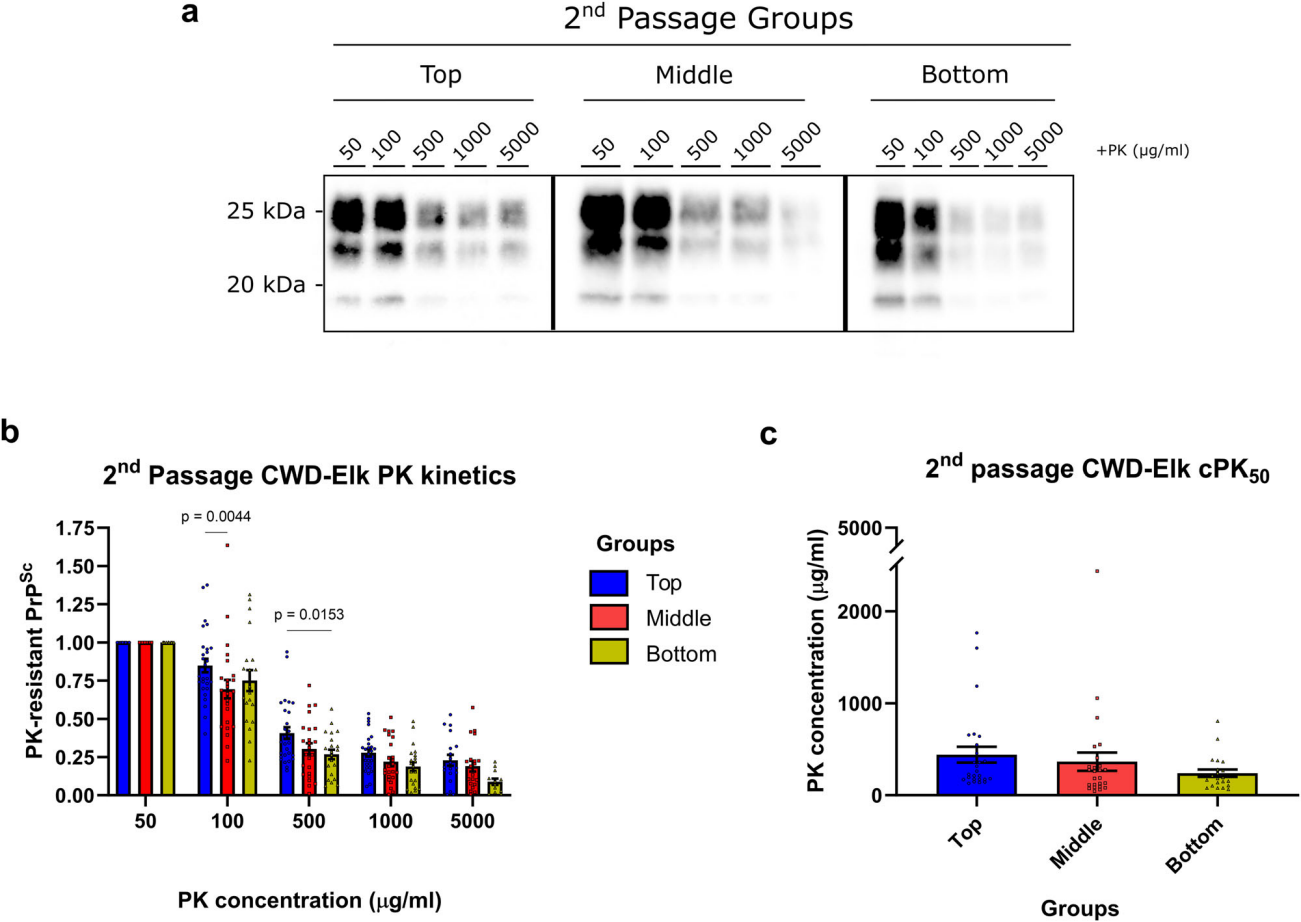
Biochemical analyses of PrP^Sc^ upon second passage in tgElk. **a** Representative Western blot of the PrP digested at various concentrations of PK from the top, middle, and bottom groups from the second passage. **b** Densitometric analysis of PrP^res^ signals. The signals were quantified as a ratio of the baseline signal at 50 μg/ml. Statistical analysis was performed with two-way ANOVA followed by Tukey’s post-hoc multiple comparison test. **c** cPK_50_ analysis of the PrP^res^ signals, with the PK concentration required to degrade 50% of the signal obtained from the baseline signal of 50 μg/ml digestion. Mean ± SEM; top group n = 21, middle group n = 25, bottom group n = 26 independent experiments with a minimum of nine biologically independent samples.

We next performed PK digestion on these second passage brain samples (Fig. 8). Even though the PrP^Sc^ in the brains of the top group was significantly more resistant at 100 and 500 μg/ml of PK than the middle and bottom groups (Fig. 8b), the cPK_50_ values for the three groups did not significantly differ from each other (Fig. 8c). However, from the first passage to the second, the cPK50 of the bottom group was significantly decreased from 1,139 ± 298 μg/ml to 240 ± 41 μg/ml (Figs. 3c and 8c; Supplementary Fig. 3b). In the top group a slight but statistically insignificant increase in cPK_50_ from 274 ± 42 μg/ml to 441 ± 86 μg/ml was observed, while the value for the middle group did not change (Supplementary Fig. 3b).

We performed ELISA with the second passage brain homogenates, with or without PK digestion, to determine the effects of passaging on the PrP^res^ and PrP^Sc^ quantity. The middle group, similar to the first passage, had lower concentrations of PrP^res^ and PrP^Sc^ in the second passage compared to the bottom and top fractions (Fig. 4a). Only in the middle group, a small but statistically significant increase in PrP^res^ was found upon passaging (Supplementary Fig. 3c). However, the most striking finding is the significant increase in PrP^Sc^ levels in all the groups, with nearly (top and bottom group) or more than twice (middle group) the amount of PrP^Sc^ in the second passage compared to the first (Supplementary Fig. 3c). This resulted in a relative increase of PrP^Sc^ compared to PrP^res^ levels, and all groups had more than twice the concentration of PrP^Sc^ compared to PrP^res^ (Fig. 4b). These data suggest that the reduction of survival in the second passage is correlated with a substantial increase of PK-sensitive PrP^Sc^.

**Fig. 9:**
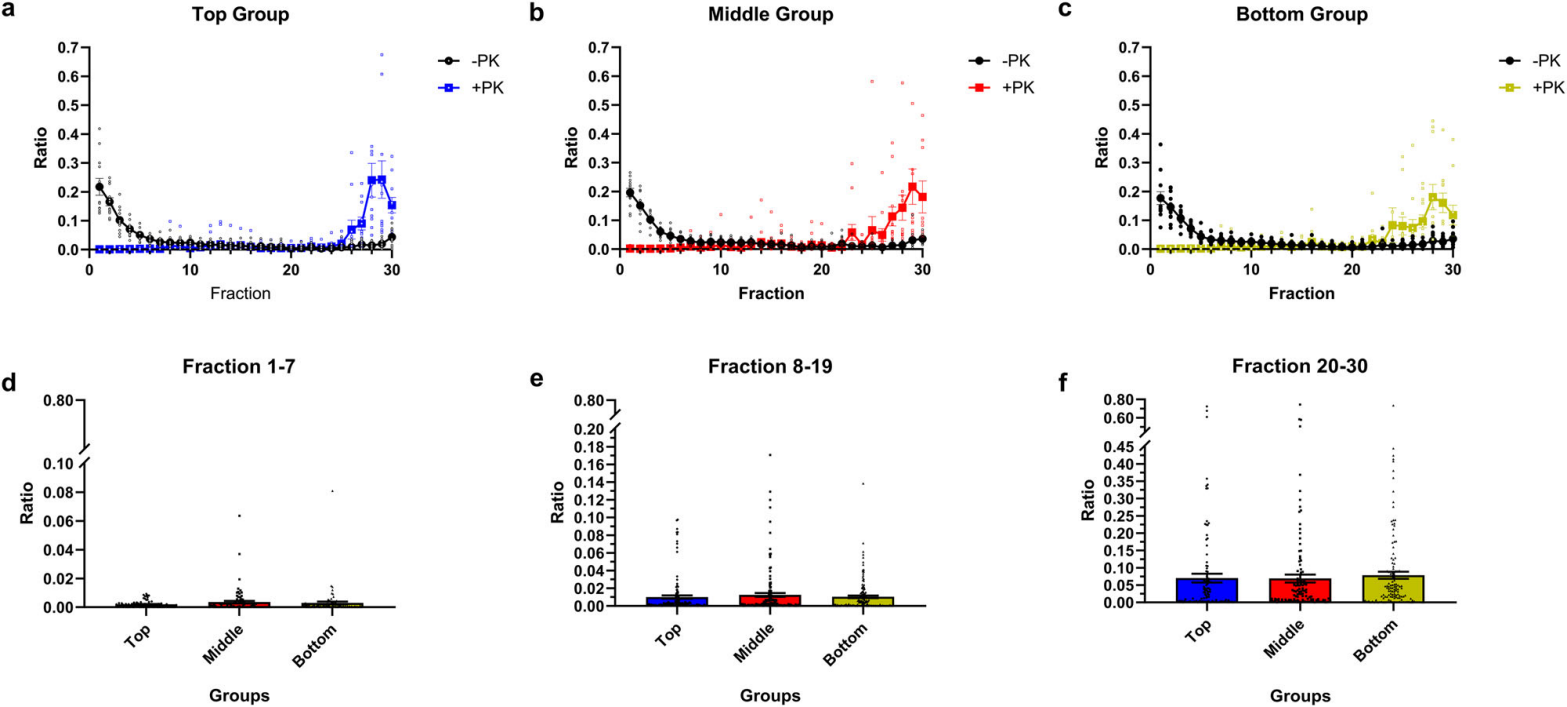
Sedimentation profiles of top, middle, and bottom groups in second passage. Second passage samples were solubilized and subjected to sedimentation velocity ultracentrifugation. Brain homogenates from top (**a**), middle (**b**), and bottom (**c**) groups were solubilized and fractionated by SV. Fractions collected from the gradients were quantified for PrP content (-PK; black line (**a**–**c**); +PK; blue (**a**), red (**b**), and green (**c**), respectively for top, middle, and bottom groups). To quantify and compare the PrP^res^ content, the +PK signals at the top (**d**), middle (**e**), and bottom (**f**) of the gradient denoted respectively as fractions 1–7, 8–19, and 20–30 were depicted as the average signal per fraction in each group. Mean ± SEM; n = 11–12 independent experiments with a minimum of four biologically independent samples.

Next, we characterized the sedimentation profiles of the prions upon second passage (Fig. 9). The profiles obtained from the three groups converged, with the highest quantity of PrP^res^ in fractions 20–30 (Fig. 9f). No discerning PrP distributions were found between the groups (Fig. 9a–f) and the sedimentation profiles (Fig. 9a–c) resembled that of the original CWD-elk prions (Fig. 2a).

The histopathological analyses of the second passage brain samples provided further insight into the biological and pathological properties manifested by the passaged prions. As in the first passage, the lesion profiles of all three groups were similar (Fig. 6c). When compared to the first passage, there were slight increases in vacuolation scoring in specific brain regions proximal or part of the diencephalon in the second passage for the middle and bottom groups, including in the parietal cortex, hippocampus, thalamus, and cerebellum for the middle group, and in the cerebellum for the bottom group. We also found that the PrP^Sc^ deposition profiles were essentially identical in the second passage between the groups, in contrast to the first passage (Fig. 6d). Specifically, the top group had more pronounced deposits in the frontal cortex, basal ganglia, and midbrain, the middle group had generally the same level of deposits except for notable decreases in the hypothalamus and cerebellum, and the bottom group had universally decreased deposits except in the frontal cortex and basal ganglia.

Altogether, upon second passage, a decrease in survival times and an increase in PrP^Sc^ levels, but not PrP^res^ was found. While PK resistance, sedimentation profiles and PrP^Sc^ distribution in the brain converged, differences in clinical signs were transmitted upon passage.

## Discussion

Prion isolates contain a continuum of structurally diverse PrP^Sc^ particles responsible for a given disease phenotype. Studies conducted so far uncovered the complexity of PrP^Sc^ particle heterogeneity and its link to strain biological properties, including infectivity and transmissibility, but nearly all have been performed on rodent-adapted prion strains^17–19,22–24^, while very little, if any, is known about prions propagated in vivo in their natural host. To our knowledge, this study is the first to investigate in detail the biophysical and biochemical properties as well as amounts of PrP^Sc^ generated in tgElk mice inoculated with elk PrP^Sc^ aggregates of different complexity upon first and second passage. Remarkably, tgElk mice inoculated with the top fractions displayed signs of hyperexcitability, animals inoculated with the bottom fractions were lethargic, and those inoculated with the middle fractions had no unique signs but had the shortest survival times although inoculated with the lowest amounts of PrP. This suggests that the survival times and pathological outcomes of prion-infected animals are not related to the amount of PrP present in the inocula, but rather linked to the quaternary structure of the prions. In addition to survival times and clinical signs, other distinctive properties were identified in each group upon first passage, (i) PK resistance, (ii) PrP^res^ and PrP^Sc^ levels, (iii) sedimentation velocity, and (iv) PrP^Sc^ deposits in different brain regions. Upon second passage, survival times were decreased in all groups, and PK resistance, PrP^Sc^ distribution and PrP^Sc^ aggregate profiles converged and resembled that found in the original elk brain homogenate. Intriguingly, the clinical signs of hyperexcitability for the top group and lethargy for the bottom group were still manifest upon passage.

PrP oligomers comprised of 14 to 28 molecules were the most infectious in one strain of hamster-adapted scrapie, 263K, while infectivity was significantly reduced in particle sizes outside this range^19^. Though the existence of PK-sensitive forms of PrP^Sc^ (PrP^sen^) has been reported^60–62^, and characterized as having the same structural features as PK-resistant PrP^Sc^ as well as the same infectivity^62^, other reports have shown that high levels of PK-resistant PrP^Sc^ are not correlated with disease^63^ or infectivity^64,65^, and that non-infectious protease-resistant PrP can be produced^65^. In contrast with previous studies where infectivity was correlated with highest PrP^res^ content for rodent-adapted scrapie strains^17,18^, our results show that the shortest survival times induced in tgElk mice did not correlate with the highest content of PrP^res^ or total PrP among the fractions used as inoculum (Figs. 2 and 4).

Similar to endpoint dilution titrations in animals, quantification of relative levels of prion seeding activity can be calculated using endpoint diluted samples, where the SD_50_ is defined as the minimum seeded amount that causes 50% of RT-QuIC reactions to be positive^66^. In the same study, using 263K scrapie strain to inoculate hamsters, the authors showed that the SD_50_ values calculated using RT-QuIC were sensitively similar to endpoint dilution bioassay^66^. Using a similar approach, we showed that survival times did correlate with the SD_50_/ng PrP, and hence, also correlated with different titers of infectivity contained in the different fractions. In addition, shortest survival times observed in mice from the middle group did not align with the highest amounts of PrP^Sc^ or PrP^res^ produced in those mice. Based on the model of prion pathogenicity proposed by Collinge and Clarke^15^, our data suggest that the hypothetical PrP^L^ played a dominant role in promoting the shortest survival in the middle group in comparison to the other groups. It indicates that non-fibrillar, oligomeric PrP^Sc^ aggregates result in the generation of higher levels of PrP^L^ compared to inoculation with smaller or more complex PrP^Sc^ aggregates from the top or bottom groups, respectively. Infectious PrP^Sc^ can be composed of both PK-sensitive and -resistant PrP^Sc^. In our study, considering that the ratio between PrP^res^ and PrP^Sc^ generated upon inoculation of the middle fractions is almost equal to 1 (Fig. 4b), this means that the PrP^sen^ content in this case did not play a role in pathogenicity. This indicates that PrP^res^ is the key component produced to an upper-limit upon which the influence of PrP^L^ takes effect, in line with a two-phased kinetics pathway of infectivity and toxicity reported in vivo^67^ and in vitro^68^.

Prion strains result from distinct conformational variations that are maintained and stabilized throughout the conversion process and even passage in different hosts^69^. However, while the overall impact of conformational variation or strain properties on disease pathogenesis and PrP^Sc^ biochemical properties is well established, less is known about the contribution of different quaternary structures to those properties. Here, we narrow down the scope of the heterogeneous assortment of PrP^Sc^ aggregates within a given CWD strain by passage in the same host. We observed that the prions in the second passage adopt a specific profile that biochemically resembled that of the middle group. In fact, cPK_50_ of the top group slightly increased while those in the bottom group significantly decreased to the level of the middle group, which stayed almost identical between first and second passage (Supplementary Fig. 3b). Remarkably, this result correlated with a survival time that was comparable between different groups (Fig. 7), where all groups had the same efficient infectivity in the second passage, found initially only within the middle group during the first passage. The sedimentation velocity profiles of different groups that were distinct in the first passage (Fig. 5) converged to be comparable between them in the second passage (Fig. 9) and resembled that of the original inocula (Fig. 2a), where larger aggregates were stabilized. This convergence in PrP^Sc^ aggregate distribution might represent changes in PrP^Sc^ quaternary structures that occur during the course of prion infection^70^. When infecting mice with a specific subset of aggregates as in our study, the kinetics of convergence might have been delayed and exceeded the survival time of the tgElk mice. This, however, provides the opportunity to investigate the contribution of individual PrP^Sc^ assemblies to the pathogenesis of prion disease more accurately. Our findings indicate that prion strains in the process of adaptation/evolution will adopt an ideal quaternary structure arrangement to gain most effective propagation, infectivity, and ultimately, toxicity. We may therefore suppose that in the context of prion ensembles, where a range of PrP molecules constitutes the infectious and toxic units that may overlap in space and time^15,67,71^, dominant prion substrains are those that most efficiently achieve this ideal assembly of different PrP^Sc^ quaternary structures distinct in their infectious or toxic nature.

It is worth noting that different particle sizes induced different clinical signs in the mice, which were retained upon second passage despite identical PrP^Sc^ biochemical and biophysical properties, vacuolation profile, and PrP^Sc^ distribution profile in the brain. There are various explanations to account for this observation, including the different local PrP^C^ expression levels and glycoform profiles, known to be key pathological determinants of prion disease biochemical and survival properties^70,72,73^, that may be differentially interacting with the different PrP^Sc^ aggregate sizes in the first passage. Alternatively, early events in the pathogenesis before biochemical and biophysical properties converged might have resulted in inheritance of clinical presentation. However, the fact that only the difference in clinical signs was retained in the second passage suggests the inclusion of other players, such as cofactors, being involved in this process. Cofactors, including endogenously expressed polyanions (such as RNA)^8,74^ and phospholipids^75^, play critical roles in guiding the infectivity of prions. In fact, it has been demonstrated that the addition of cofactors to either native PrP^C^^76^ or recombinant PrP^77^ in PMCA is enough to generate infectious prions. Furthermore, prion replication environment plays an important role in defining the fate of prion strain adaptation^78^, and different prion strains with intrinsically different neuropathological and conformational stability profiles, when propagated in the presence of the same purified cofactors, had such properties converge^79^.

In the context of our study, we propose that the different aggregation states assumed by the fractionated prions used for the inoculation during the first passage affected the selection and interactions of cofactors with the PrP^Sc^ seeds and guided replication to brain areas where these co-factors are available. This can explain the difference in PrP^Sc^ distribution in some brain regions in the first passage. In the absence of fractionation, the CWD prions in tgElk would predominantly interact with an ideal mix of co-factors. Upon fractionation, their ability to do this was restricted to a certain subset of co-factors specific to each fraction, and this consequently yields reduced infectivity, potentially delaying the production of PrP^L^ resulting in extended survival times, and different prion properties in the first passage among the groups. We suggest that the selection of a given cofactor and brain region for replication is associated with the specific clinical signs i.e., lethargy or hyperexcitability. In the second passage, different clinical signs were retained but PrP^Sc^ distribution converged at the terminal stage. There are two possible explanations: either the association with a specific co-factor is a dominant and transmissible determinant of clinical outcome, in line with previous studies^78,80,81^, or at the early stages of infection with a predominant presence of a specific PrP^Sc^ aggregate subset replication and toxicity is first occurring in the same brain regions as in the first passage. Only at later stages in the pathogenesis, a balanced mixture of PrP^Sc^ aggregates is formed and distribution of those converges. One option to verify this hypothesis is to replicate the second passage and perform kinetic studies of PrP^Sc^ distribution throughout preclinical and clinical stages of disease.

While the material we used for fractionation has been passaged in elk and characterized as the CWD2 prion strain^48^, we did not use a biologically cloned strain. Therefore, we cannot rule out that it contained a mixture of co-existing prion strains, where a dominant strain, here CWD2, suppresses the propagation of non-dominant strains upon inoculation of whole brain homogenate. By contrast, PrP^Sc^ aggregates of dominant and non-dominant strains may be enriched in different fractions upon sedimentation velocity centrifugation. In this scenario, the differences observed between the groups in the first passage might be attributable to infection with distinct substrains. However, most of the observed differences in biochemical, neuropathological, and sedimentation properties, converged between the groups upon passaging. This contrasts with the transmissibility and conservation of those properties that define strains; therefore, we exclude a major influence of substrains. Furthermore, a recent study demonstrated that even biologically cloned strains can contain suppressed substrains^82^, suggesting that strain mixtures cannot be ruled out even if we used biologically cloned strains.

In summary, we suggest that PrP^Sc^ aggregate size or quaternary structure is an important determinant of the production of toxic PrP species, clinical outcome and PrP^Sc^ distribution in the brain, possibly by mediating co-factor interactions.

## Methods

### Ethics

We have complied with all relevant ethical regulations for animal use. This study followed the guidelines of the Canadian Council for Animal Care, and all animal experiments in the study were approved by the University of Calgary Animal Care Committee under protocol number AC18-0047.

We used isoflurane at a concentration of 5% (flow rate of 0.8 L/min) for induction as anesthesia, and then we lowered the concentration to 0.5–1% for maintenance of general anesthesia prior to all inoculations. Isoflurane overdose was performed for euthanasia.

### CWD prions

CWD-Elk samples were prepared at a final concentration of 10% (wt/vol.) in phosphate-buffered saline (PBS; Life Technologies, Gibco) from harvested brain samples using either a dounce homogenizer or the MP Biomedicals fast prep‐24 homogenizer (Fisher). Aliquots were stored at −80 °C for further use. The CWD-Elk prion sample was a pool of brain homogenates of three elk experimentally inoculated via oral route with brain homogenate from a CWD-positive, farmed elk^56^ (kindly provided by Dr. Stefanie Czub, Canadian Food Inspection Agency, Lethbridge – Canada). This pool has been characterized as strain CWD2 by Angers and colleagues, referred to as elk ‘Alberta CWD pool’^48^.

### Sedimentation velocity gradient

The procedure for the sedimentation velocity ultracentrifugation of the prion samples was performed with slight modifications, as indicated below, to protocols published by Tixador et al. and Laferrière et al.^17,18^. Briefly, 100 μl of 10% brain homogenate was solubilized in an equal volume of solubilization buffer (50 mM HEPES pH 7.4, 300 mM NaCl, 10 mM EDTA, 2 mM DTT, 4% (wt/vol.) dodecyl-β-D-maltoside; Sigma) on ice for 45 min. Sarkosyl (N-lauryl sarcosine; Sigma) was added to a final concentration of 2% (wt/vol.) and the incubation continued for another 45 min on ice. The sample was loaded onto a continuous 10-25% iodixanol gradient (Optiprep; Sigma), the linearity of which was verified by refractometry. The gradients were centrifuged at 285,000 *g* for 45 min at 4 °C in a SW-55 rotor using an Optima XE-90 ultracentrifuge (Beckman Coulter). Applying such high relative centrifugal force enables the separation of molecules according to molecular mass and shape. Gradients were separated into 30 equal fractions of 160 μl from the top. Standard markers (GE Healthcare) consisting of lactate dehydrogenase (140 kDa), catalase (232 kDa), ferritin (440 kDa), and thyroglobulin (669 kDa) were run as controls^17^.

### Mouse Bioassay

Female tgElk mice between six to eight weeks old were anesthetized and intracerebrally inoculated with 20 μl of the fractions obtained upon sedimentation velocity centrifugation, or 1% BH (vol./vol. in PBS) sample in the right parietal lobe using a 25-gauge disposable hypodermic needle. The mice were monitored weekly until the onset of clinical signs, at which point they were monitored daily. At the experimental endpoint when mice reached terminal prion disease, the mice were anesthetized before being euthanized by isofluorane overdose. Brain samples from these animals were collected and either frozen at −80 °C or fixed in 10% formalin for further analyses.

### PK digestion

Fractions were digested with 10 μg/ml of PK (Roche) at 37 °C for 1 h, and 1x Pefabloc protease inhibitor (VWR) was added to terminate the enzymatic reaction. Five volumes of methanol (Fisher) were added into each tube that were then kept at −20 °C for at least 1 h to precipitate the proteins. Then, the samples were centrifuged at 21,130 *g* for 2.5 h to pellet the protein, after which the methanol was discarded, and the protein pellet was resuspended in 3X sample loading buffer and subjected to SDS-PAGE and Western blot. At least three replicates were performed.

To determine the cPK_50_, brain homogenates were digested with a range of PK concentrations from 50 μg/ml to 5000 μg/ml at 37 °C for 1 h, and 1x Pefabloc protease inhibitor (VWR) was added to terminate the enzymatic reaction, followed by SDS-PAGE and Western blot. There were at least three brain samples when possible, representing each fraction, and at least three replicates were performed.

### SDS-PAGE and western blot

Samples were denatured at 95 °C for 10 min in 3X SDS sample buffer. They were separated on a 12.5% SDS-poly-acrylamide gel, and then electrophoretically transferred to PVDF membranes (Amersham, GE Healthcare). Membranes were probed with the anti-PrP monoclonal antibody 4H11^83^ (1:500) followed by horseradish peroxidase-conjugated goat anti-mouse IgG antibody (1:5000; Sigma) and developed using Luminata horseradish peroxidase substrate (MilliporeSigma). Images were acquired on either autoradiography films (Super Rx; Fujifilm; Denville Scientific) or digital imaging systems (FluorChemQ (Alpha Innotech); ChemiDoc (Bio-Rad)). ImageJ was used to quantify the PrP signals on the autoradiography films, while the FluorChemQ (Alpha Innotech) or the Image Lab (Bio-Rad) software were used to quantify the PrP signals on the respective digital images. Calculations were done in Microsoft Excel.

### ELISA

Detection of PrP^Sc^ and PrP^res^ was performed following the protocol in Hannaoui et al.^51^. Briefly, PrP^Sc^ or PrP^res^ were enriched by incubating a 50 µl aliquot of brain homogenates with or without PK-treatment in 1.8 ml of RiPa cell lysis buffer (150 mM NaCl, 1% NP-40, 0.25% Sodium deoxycholate, 1 mM EDTA, 50 mM Tris, pH 7.4) at room temperature for 30 min. After centrifugation for 20 min at 20,000 *g*, the sample separates into an insoluble pellet fraction. The pellet was re-suspended in 50 μl of 8 M guanidine hydrochloride (GdnHCl). Reference samples with standardized concentrations of PrP were generated using purified recombinant mouse PrP23 – 231 (recMoPrP) which was 2-fold serially diluted in 8 M GdnHCl ranging from 2.5 to 0.039 µg/ml. These seven different concentrations of recMoPrP were used as PrP standard calibration samples and assayed by adding 5 µl each standard in 150 µl reaction buffer (1% BSA in PBS) for each ELISA test-well. Then, ELISA strip plates (Santa Cruz, USA) were coated with 0.5 µg/well of a novel anti-PrP monoclonal antibody (mAb) D15.15 (aa175 – 179) (Tang et al., manuscript in preparation) in PBS by overnight incubation at 4 °C. After blocking and washing with TBST (TBS with 0.1% of tween 20), five microliters of PrP^Sc^ or PrP^res^ enriched samples and PrP standard were mixed with 150 µl of reaction buffer and added into the test-well in triplicate. The plate was incubated for 90 min at RT, washed with TBST, and the captured PrP was detected by incubation with an HRP-conjugated anti-PrP mAb N5 (aa 97–100) (Tang et al., manuscript in preparation) for 1 h at RT. The plates were again washed with TBST and 100 µl of TMB substrate (Surmodics, USA) was added. After incubation for 20 min at RT in the dark, the absorbance was measured at 650 nm and used for PrP quantification. The average standard calibration OD values obtained by ELISA were used to plot an 8-parameter linear curve fit to the standards and then calculate the PrP concentrations for the test samples.

### Vacuolation scoring

Sagittal brain sections (4.5 μm-thick) of formalin-fixed and paraffin-embedded brain tissues were stained using hematoxylin and eosin (Leica). Spongiform degeneration was scored at nine different regions of the brain (frontal cortex, basal ganglia, parietal cortex, hippocampus, thalamus, hypothalamus, midbrain, cerebellum, and medulla/pons) on a scale of 0 (absence) to 5 (severe) for the presence and severity of spongiform degeneration. The scoring was performed at least 6 times in a blinded manner.

### Immunohistochemistry

Serial sections of formalin-fixed and paraffin-embedded brains were cut at 5 μm thickness. They were autoclaved (2.1 × 10^5 ^Pa) for 30 min in citric acid (10 mM), pH 6.0, at 121 °C, followed by incubation in 98% formic acid (Sigma) for 30 min, then 4 M guanidine thiocyanate (Sigma) for 2 h. Abnormal PrP accumulation was examined using a commercially available ARK (Animal Research Kit)/HRP kit (DAKO) by using the anti-PrP monoclonal antibody 12B2 (aa 93–97; 1:800; Wageningen Bioveterinary Research) for 30 min at 37 °C and sections were counterstained with hematoxylin. Slides were scanned using the Olympus VS110-5S scanner, and images were analyzed using OlyVIA software (Olympus). PrP^Sc^ distribution was scored at nine different regions of the brain (frontal cortex, basal ganglia, parietal cortex, hippocampus, thalamus, hypothalamus, midbrain, cerebellum, and medulla) on a scale of 0 (absence) to 5 (severe).

### Protein misfolding cyclic amplification (PMCA)

PMCA was performed using protocol by Arifin et al.^84^, with some modifications as indicated below. Briefly, brains from non-inoculated tgElk mice were prepared as 10% (w/v) BH using a Potter–Elvehjem PTFE pestle and glass tube (Sigma-Aldrich, #P7984) in cold PMCA conversion buffer containing 4 mM EDTA, 1% Triton X-100 and 1 tablet cOmplete™ Protease Inhibitor Mini (Roche) in 1× PBS and adjusted to a pH of 7.4. Samples were centrifuged at 13,000 rpm for 1 min to remove insoluble debris. The homogenates were aliquoted in sterile 2 ml tubes and stored at −80 °C until further use. Ninety microliters of substrate were added to 0.2 ml tubes (ThermoFisher, #AB0337) with three PTFE balls (McMaster-Carr, #9660K12, 3/32 in ø). Fractionated materials were diluted in 10-fold serial dilutions ranging from 10^−1^ to 10^−3^ in PMCA substrate. 10 µl of each seed dilution was added, with a final seed concentration of 10^−2^ to 10^−4^ in each reaction tube. All reactions were prepared in duplicate. Tubes were sealed with parafilm, decontaminated in 2.5 % bleach for 5 min, rinsed with H_2_O, and centrifuged briefly before they were placed in a tube rack inside a microplate sonicator horn assembly (431MPXH and Q700, QSonica) connected to a circulating water bath (CC304-B, Huber). The sonicator was set for 30 s of sonication at 375–395 W followed by 14.5 min rest and run for 24 h for a total of 96 sonication-rest cycles, corresponding to one round of PMCA. Ten microliters of the resulting sample were transferred into 90 µl of fresh PMCA substrate and run for another 24 h with the same settings to constitute serial passaging. This process was repeated to obtain three rounds of serial PMCA.

### Real-time quaking-induced conversion (RT-QuIC)

Recombinant prion protein (rPrP) with the full-length mouse prion protein sequence (aa 23–231) was used here^85^. The RT-QuIC reaction mastermix contained 10 µg of rPrP, 10 µM Thioflavin-T (ThT), 170 mM NaCl, 1× PBS (contains 130 mM NaCl), 1 mM EDTA and H_2_O. Ninety-eight microliters of the RT-QuIC mastermix was added into each well of a black 96-well plate (Corning Costar #3603) followed by 2 µl of seed. The seeds used were fractions diluted in a buffer containing 0.1% SDS with dilutions ranging from 10^−1^ to 10^−5^ and set up in four replicates. Plates were sealed with a sealing tape, placed into a BMG Labtech FLUOstar™ plate reader, and set for shaking and incubation intervals of 1-min shaking (700 rpm) and 1-min rest. The readings were plotted as the average relative fluorescence unit (RFU) against time in hours. The seeding dose/dilution at which 50% of the wells turned positive (SD_50_) was determined according to a modified Spearman-Karber analysis^66,86^, as detailed below. Wells were considered to be positive when the fluorescence reached a cutoff^85^. SD_50_ values were calculated according to the equation ${{SD}}_{50}={{antilog}}_{10}(\mid({x}_{p=1}+0.5-\mathop{\sum }\nolimits_{{x}_{p=1}}^{{x}_{\min }}{p}_{x})\mid)$, and standard error according to the equation ${SE}=\frac{1}{{\log }_{10}e}\big(\sqrt{\sum \frac{p(1-p)}{n-1}}\big)({{SD}}_{50})$, where x_p=1_ is the log_10_ dilution for which all wells were positive, x_min_ is the highest dilution used, p_x_ is the proportion positive at each respective dilution, p is the proportion positive, e is the Euler’s number, and n is the number of replicates.

### Statistics and reproducibility

All graphs and statistical calculations were generated using Graphpad Prism 9. Unless otherwise specified, statistical analysis with one-way ANOVA followed by *post-hoc* analysis with Tukey’s multiple comparison test was performed. A minimum of three replicates, using at least three biologically independent samples, were used to procure the results.

### Reporting summary

Further information on research design is available in the Nature Portfolio Reporting Summary linked to this article.

### Supplementary information

Supplementary Information

Description of Additional Supplementary Files

Supplementary Data 1

Reporting Summary

## Data Availability

Numerical source data is available in Supplementary Data 1. Uncropped and unedited Western blot membrane scans are available in Supplementary Figs. 4–7. Other relevant data may be obtained from the corresponding author upon reasonable request.
