# Supplementary material for: Propagation of PrPSc in mice reveals impact of aggregate composition on prion disease pathogenesis
Source: Commun Biol. 2023 Nov 14;6:1162. doi: 10.1038/s42003-023-05541-3 (PMC10645910; doi:10.1038/s42003-023-05541-3)
Supplement: Supplementary file 1 — Supplementary Information [file 42003_2023_5541_MOESM1_ESM.pdf]

Supplementary Information

Propagation of PrPSc in mice reveals impact of aggregate composition on prion disease pathogenesis

Sheng Chun Chang[1,2*], Samia Hannaoui[1,2*], Maria Immaculata Arifin[1,2], Yuan-Hung Huang[1,2], Xinli Tang[3], Holger Wille[3,4], Sabine Gilch[1,2+]

*These authors contributed equally

[1] Dept. of Comparative Biology and Experimental Medicine, Faculty of Veterinary Medicine, University of Calgary, Calgary, Canada

[2] Hotchkiss Brain Institute, Cumming School of Medicine, University of Calgary, Calgary, Canada

[3] Department of Biochemistry, Center for Prions and Protein Folding Diseases, University of Alberta, Edmonton, Canada

[4] Neuroscience and Mental Health Institute, University of Alberta, Edmonton, Canada

[+]corresponding author: sgilch@ucalgary.ca

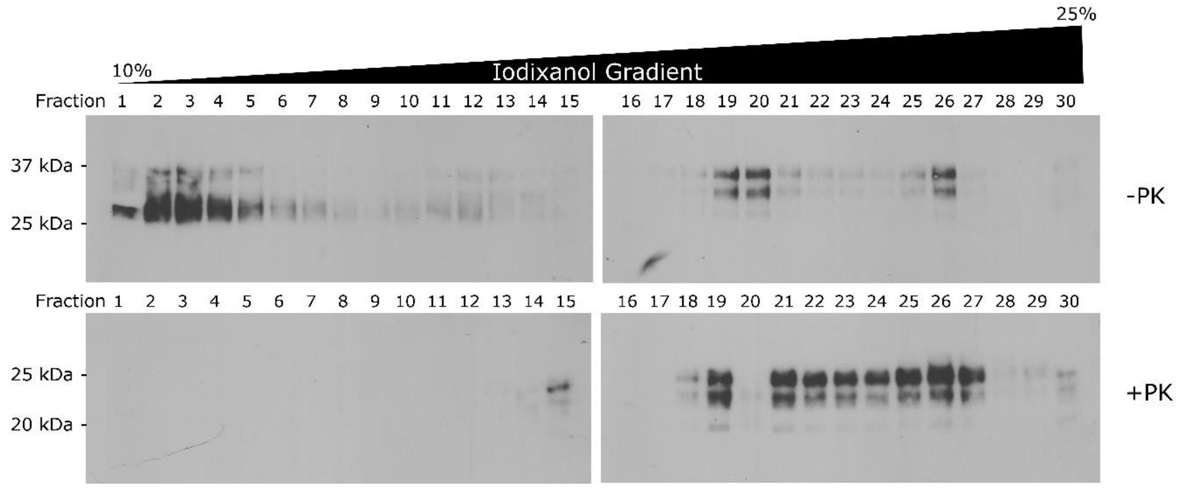

Supplementary Figure 1. Sedimentation velocity gradient of CWD-Elk fractions. (a) Representative western blot of the total PrP (top) and PrP^{res} (bottom) content depicting the total PrP and PrP^{res} of CWD-Elk prions solubilized and fractionated through sedimentation velocity ultracentrifugation. Total PrP content were greater at the top of the gradient, while PrP^{res} content were greater at the bottom of the gradient.

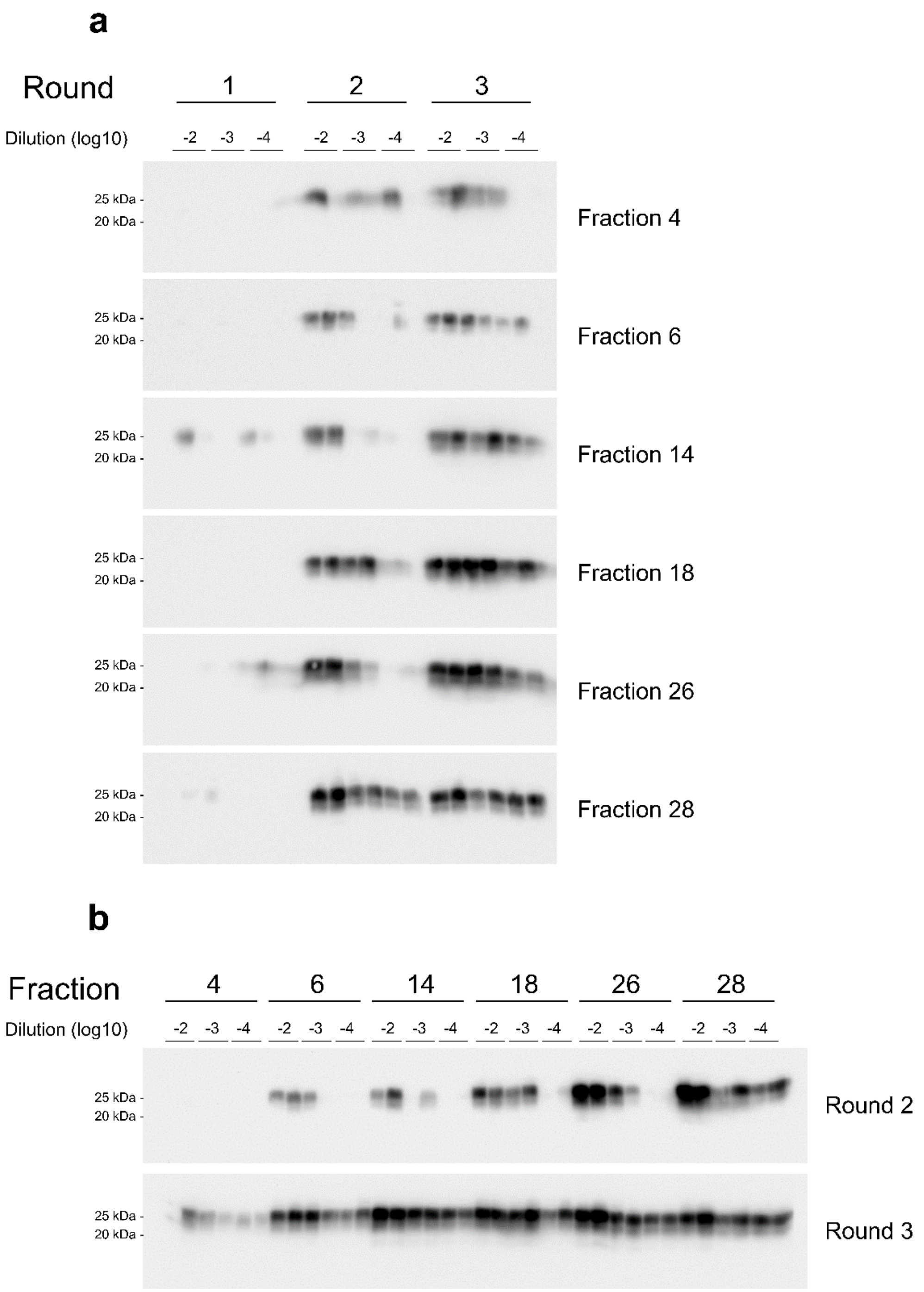

Supplementary Figure 2. Serial PMCA of fractionated CWD-elk brain homogenate. Representative fractions were serially diluted and subjected to 3 rounds of PMCA. PrP^{res} generated in each round was analysed by western blot using anti-PrP mAb 4H11. (a) PMCA products generated over 3 rounds of PMCA from the specific fractions were compared. (b) Amount of PrP^{res} was compared in each round between the different fractions.

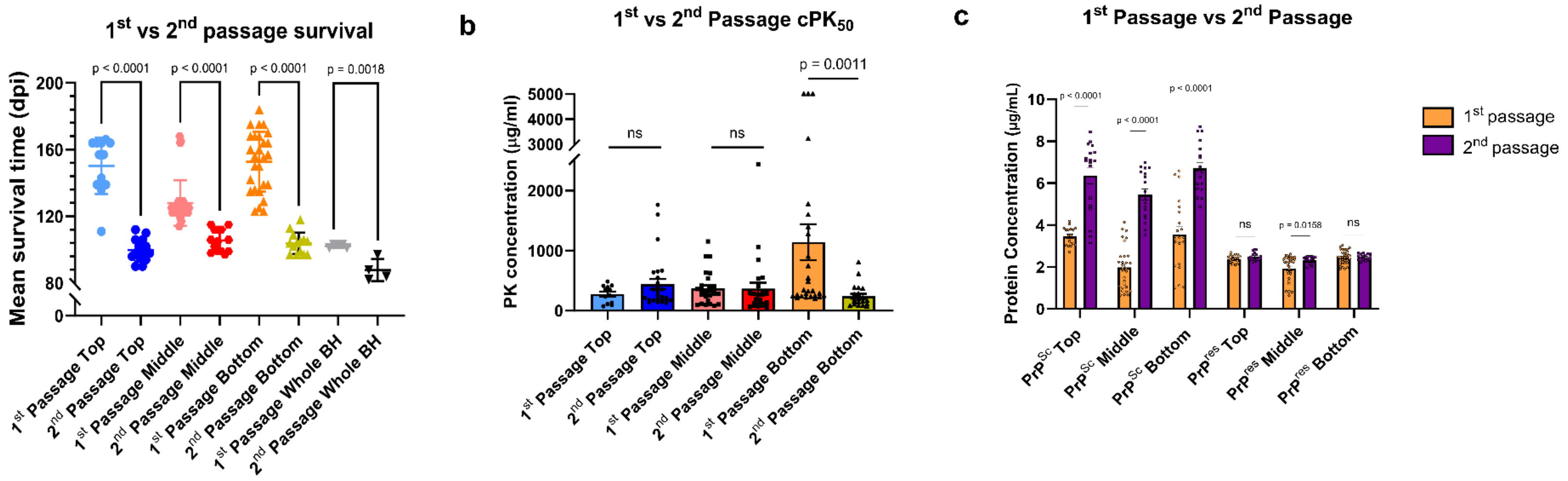

Supplementary Figure 3. Comparison of first and second passage of fractionated prions in tgElk. (a) Depiction of the survival, with an approximate 50-day decrease in the top and bottom groups and 20-day decrease in the middle group from the first to the second passage. (b) Depiction of the cPK_{50}, with an insignificant increase in the top group, no change in the middle group, and a significant decrease in the bottom group through passaging. (c) Depiction of the ELISA, with a drastic increase in PrP^{Sc} levels across all groups, and a slight PrP^{res} increase observed in the middle group, from first to second passage. Statistical analyses were performed with unpaired Student's *t*-test.

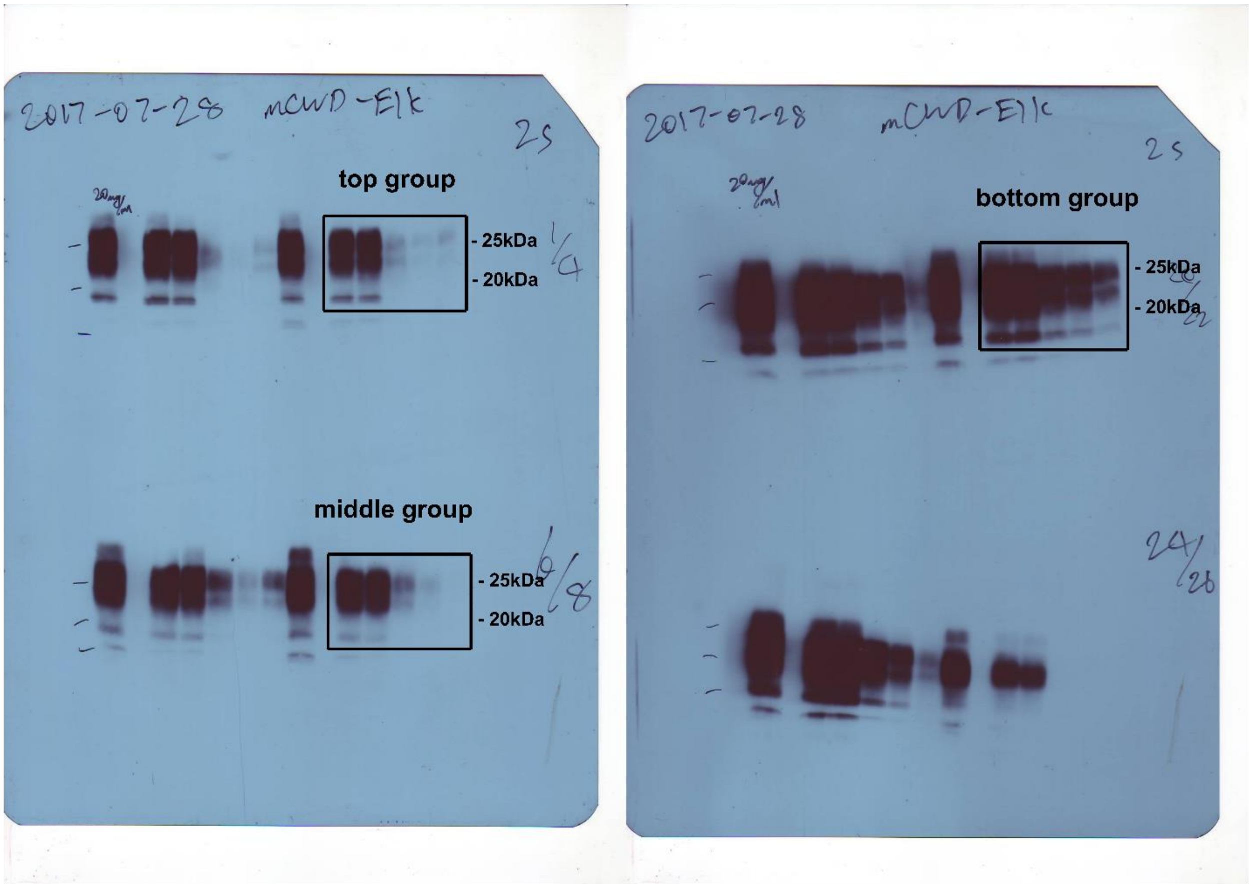

Supplementary Figure 4. Uncropped and unedited western blot of Fig. 3a.

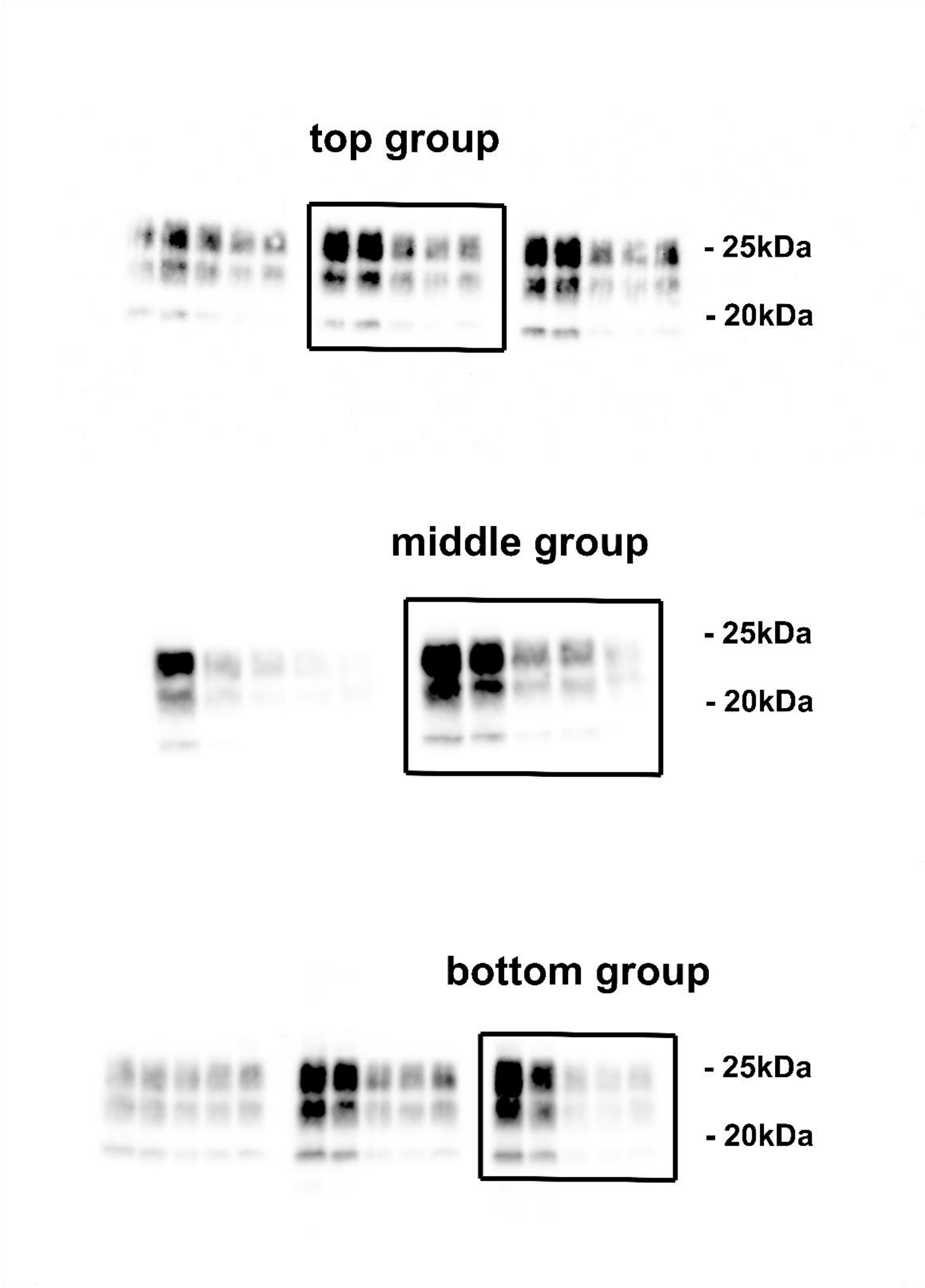

Supplementary Figure 5. Uncropped and unedited western blot of Fig. 8a.

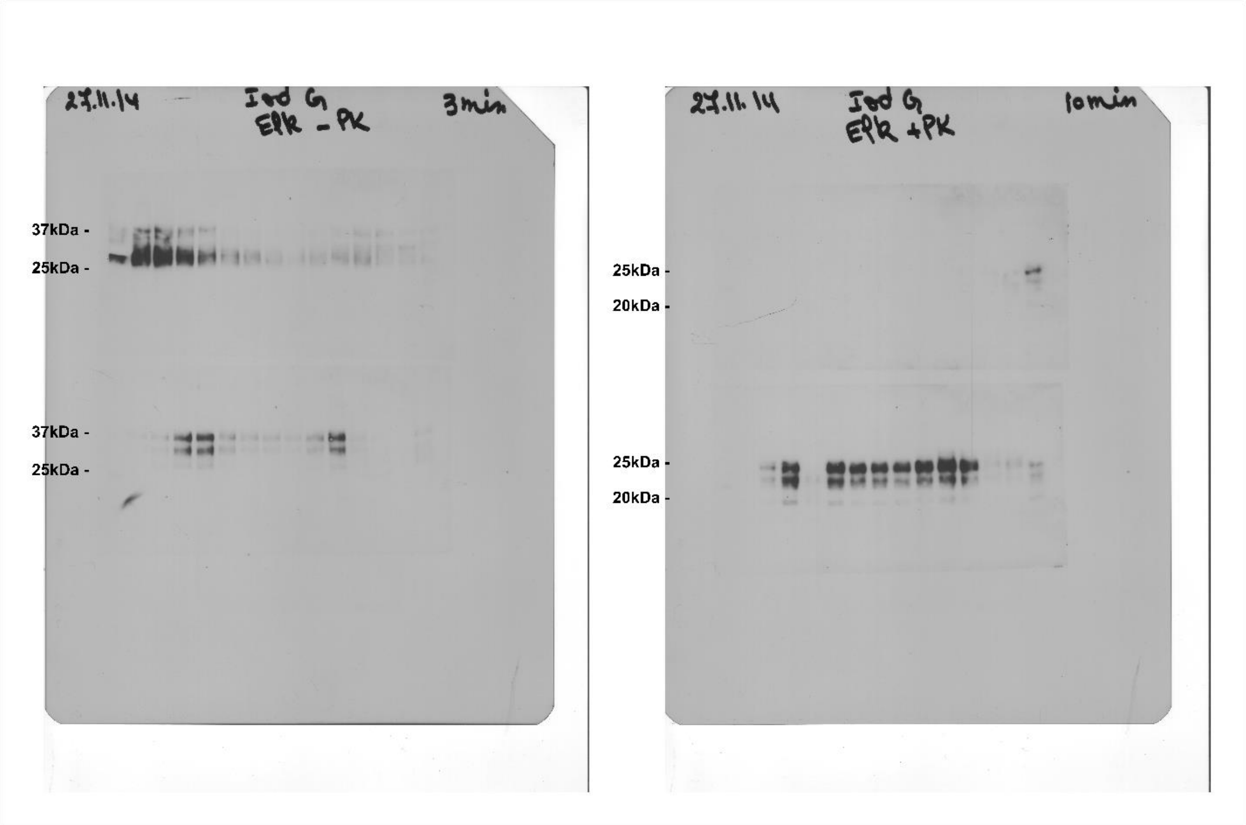

Supplementary Figure 6. Uncropped and unedited western blot of Supplementary Fig. 1.

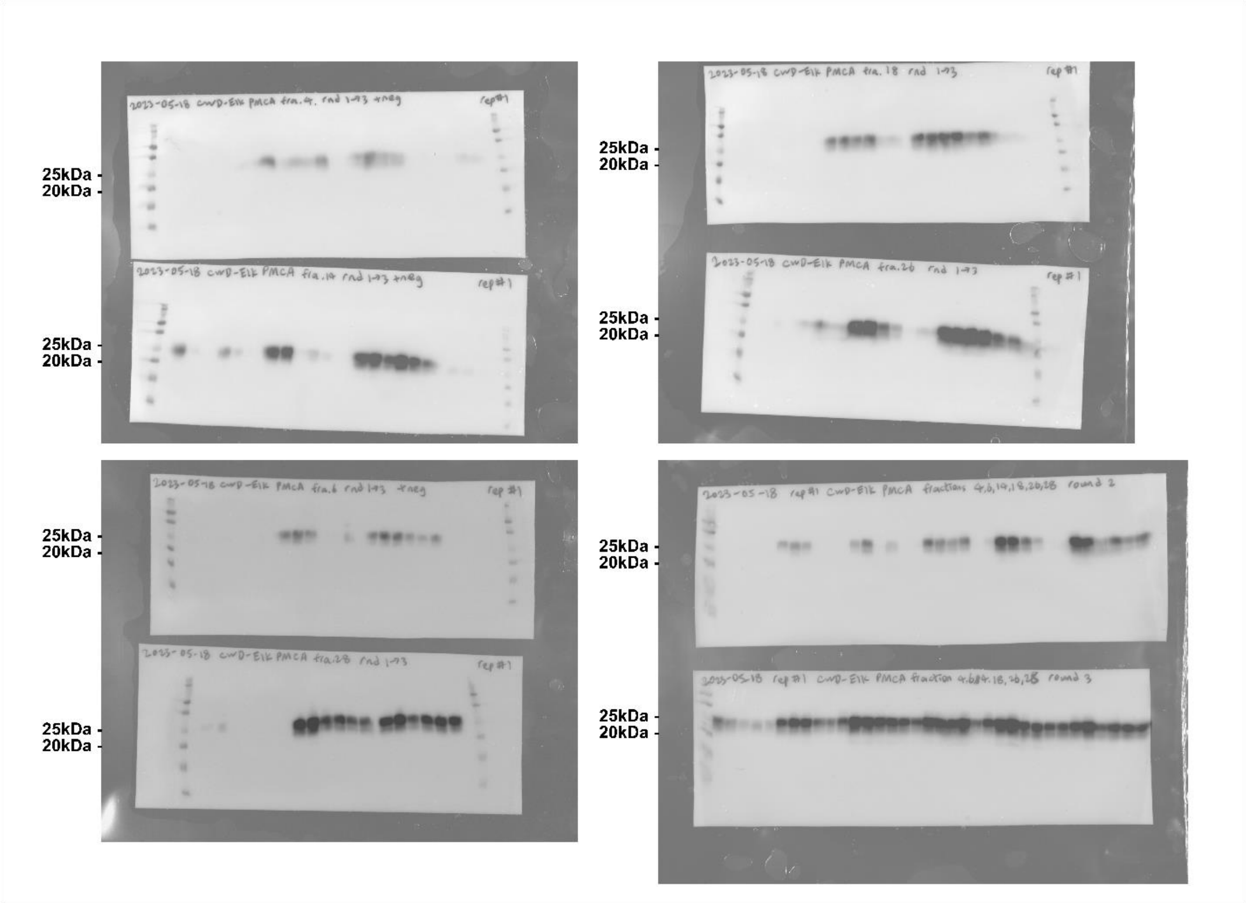

Supplementary Figure 7. Uncropped and unedited western blot of Supplementary Fig. 2.
