# Supplementary material for: Propagation of PrPSc in mice reveals impact of aggregate composition on prion disease pathogenesis
Source: Commun Biol. 2023 Nov 14;6:1162. doi: 10.1038/s42003-023-05541-3 (PMC10645910; doi:10.1038/s42003-023-05541-3)
Supplement: Supplementary file 2 — Description of Additional Supplementary Files [file 42003_2023_5541_MOESM2_ESM.pdf]

Description of Additional Supplementary Files

File name: Supplementary Data 1
Description: Numerical source data behind the graphs in the figures.
