# Supplementary material for: Propagation of PrPSc in mice reveals impact of aggregate composition on prion disease pathogenesis
Source: Commun Biol. 2023 Nov 14;6:1162. doi: 10.1038/s42003-023-05541-3 (PMC10645910; doi:10.1038/s42003-023-05541-3)
Supplement: Supplementary file 4 — Reporting Summary [file 42003_2023_5541_MOESM4_ESM.pdf]

nature portfolio

Corresponding author(s): Sabine Gilch

Last updated by author(s): Oct 18, 2023

Reporting Summary

Nature Portfolio wishes to improve the reproducibility of the work that we publish. This form provides structure for consistency and transparency in reporting. For further information on Nature Portfolio policies, see our Editorial Policies and the Editorial Policy Checklist.

Statistics

For all statistical analyses, confirm that the following items are present in the figure legend, table legend, main text, or Methods section.

n/a	Confirmed	
☐	☒	The exact sample size (*n*) for each experimental group/condition, given as a discrete number and unit of measurement
☐	☒	A statement on whether measurements were taken from distinct samples or whether the same sample was measured repeatedly
☐	☒	The statistical test(s) used AND whether they are one- or two-sided<br>*Only common tests should be described solely by name; describe more complex techniques in the Methods section.*
☒	☐	A description of all covariates tested
☐	☒	A description of any assumptions or corrections, such as tests of normality and adjustment for multiple comparisons
☐	☒	A full description of the statistical parameters including central tendency (e.g. means) or other basic estimates (e.g. regression coefficient) AND variation (e.g. standard deviation) or associated estimates of uncertainty (e.g. confidence intervals)
☐	☒	For null hypothesis testing, the test statistic (e.g. *F*, *t*, *r*) with confidence intervals, effect sizes, degrees of freedom and *P* value noted<br>*Give P values as exact values whenever suitable.*
☒	☐	For Bayesian analysis, information on the choice of priors and Markov chain Monte Carlo settings
☒	☐	For hierarchical and complex designs, identification of the appropriate level for tests and full reporting of outcomes
☒	☐	Estimates of effect sizes (e.g. Cohen's *d*, Pearson's *r*), indicating how they were calculated

Our web collection on statistics for biologists contains articles on many of the points above.

Software and code

Policy information about availability of computer code

Data collection	*Provide a description of all commercial, open source and custom code used to collect the data in this study, specifying the version used OR state that no software was used.*
Data analysis	*Provide a description of all commercial, open source and custom code used to analyse the data in this study, specifying the version used OR state that no software was used.*

For manuscripts utilizing custom algorithms or software that are central to the research but not yet described in published literature, software must be made available to editors and reviewers. We strongly encourage code deposition in a community repository (e.g. GitHub). See the Nature Portfolio guidelines for submitting code & software for further information.

Data

Policy information about availability of data

All manuscripts must include a data availability statement. This statement should provide the following information, where applicable:

- Accession codes, unique identifiers, or web links for publicly available datasets
- A description of any restrictions on data availability
- For clinical datasets or third party data, please ensure that the statement adheres to our policy

Numerical source data is available in Supplementary Data 1. Uncropped and unedited western blot membrane scans are available in Supplementary Figures 4 – 7. Other relevant data may be obtained from the corresponding author upon reasonable request.

Human research participants

Policy information about studies involving human research participants and Sex and Gender in Research.

Reporting on sex and gender	*Use the terms sex (biological attribute) and gender (shaped by social and cultural circumstances) carefully in order to avoid confusing both terms. Indicate if findings apply to only one sex or gender; describe whether sex and gender were considered in study design whether sex and/or gender was determined based on self-reporting or assigned and methods used. Provide in the source data disaggregated sex and gender data where this information has been collected, and consent has been obtained for sharing of individual-level data; provide overall numbers in this Reporting Summary. Please state if this information has not been collected. Report sex- and gender-based analyses where performed, justify reasons for lack of sex- and gender-based analysis.*
Population characteristics	*Describe the covariate-relevant population characteristics of the human research participants (e.g. age, genotypic information, past and current diagnosis and treatment categories). If you filled out the behavioural & social sciences study design questions and have nothing to add here, write "See above."*
Recruitment	*Describe how participants were recruited. Outline any potential self-selection bias or other biases that may be present and how these are likely to impact results.*
Ethics oversight	*Identify the organization(s) that approved the study protocol.*

Note that full information on the approval of the study protocol must also be provided in the manuscript.

Field-specific reporting

Please select the one below that is the best fit for your research. If you are not sure, read the appropriate sections before making your selection.

☒ Life sciences ☐ Behavioural & social sciences ☐ Ecological, evolutionary & environmental sciences

For a reference copy of the document with all sections, see nature.com/documents/nr-reporting-summary-flat.pdf

Life sciences study design

All studies must disclose on these points even when the disclosure is negative.

Sample size	There was no sample-size calculation performed. We have used 5 mice per group, which was the minimum required to have relevant statistical analyses. We also planned on pooling different groups of mice, which provided us with a minimum of 15 mice within a pool to a maximum of 30 mice. The number of animals we used was also bound by our animal protocols (3Rs).
Data exclusions	Some animals were excluded from the analyses. The exclusion criteria were pre-established and they included animals not reaching an experimental endpoint, but rather, dying of other reasons (e.g. an intercurrent disease causing the animal to reach a humane endpoint)
Replication	Reproducibility was verified with biological and experimental replicates. We performed a minimum of three replicates for all experiments
Randomization	No randomization was performed to allocate subjects in the study. It is not relevant to our study as we use an inbred mouse line with mice in the same range of age and of same sex.
Blinding	All animal experiments were blinded to the experimenter during the animal experimentation. Animals are checked by two persons that rotate, while there was no blinding for statistical analysis.

Reporting for specific materials, systems and methods

We require information from authors about some types of materials, experimental systems and methods used in many studies. Here, indicate whether each material, system or method listed is relevant to your study. If you are not sure if a list item applies to your research, read the appropriate section before selecting a response.

Materials & experimental systems

n/a	Involved in the study
☐	☒ Antibodies
☒	☐ Eukaryotic cell lines
☒	☐ Palaeontology and archaeology
☐	☒ Animals and other organisms
☒	☐ Clinical data
☒	☐ Dual use research of concern

Methods

n/a	Involved in the study
☒	☐ ChIP-seq
☒	☐ Flow cytometry
☒	☐ MRI-based neuroimaging

Antibodies

Antibodies used	- 4H11: (Ref: Ertmer, A. et al. The tyrosine kinase inhibitor STI571 induces cellular clearance of PrPSc in prion-infected cells. J. Biol. Chem. 279, 41918–41927 (2004))<br>- HRP peroxidase-conjugated goat anti-mouse IgG: Sigma, product A9917<br>- D15.15 (Ref: Tang et al., manuscript in preparation)<br>- 12B2: (Wageningen Bioveterinary Research; Ref: Langeveld et al. Rapid and discriminatory diagnosis of scrapie and BSE in retro-pharyngeal lymph nodes of sheep. BMC Vet. Res. 2006, 2:19)
Validation	All data and references are provided within the manuscript or provided to reviewers for confidential evaluation

Animals and other research organisms

Policy information about studies involving animals; ARRIVE guidelines recommended for reporting animal research, and Sex and Gender in Research

Laboratory animals	- Mouse line (tgElk)<br>- Four to six weeks of age at the time of inoculation
Wild animals	The study did not involve wild animals
Reporting on sex	The findings apply to only one sex.<br>Data disaggregation for sex has not been collected.<br>Only female mice were used to prevent serious fighting between male mice.
Field-collected samples	The study did not involve samples collected from the field
Ethics oversight	University of Calgary Animal Care Committee

Note that full information on the approval of the study protocol must also be provided in the manuscript.
